# Effect of fat distribution on left ventricular structure and function in different sexes: a Mendelian randomization study

**DOI:** 10.3389/fendo.2025.1355968

**Published:** 2025-02-25

**Authors:** Hang Li, Guangjiao Yin, Yanfang Zhang, Ziwei Wang, Fang Lv, Rui Li, Juanjuan Qin, Xujun Ye

**Affiliations:** ^1^ Department of Geriatrics, Zhongnan Hospital of Wuhan University, Wuhan, Hubei, China; ^2^ Zhongnan Hospital of Wuhan University, Institute of Hepatobiliary Diseases of Wuhan University, Transplant Center of Wuhan University, Wuhan, Hubei, China; ^3^ Center for Healthy Aging, Wuhan University School of Nursing, Wuhan, Hubei, China

**Keywords:** fat distribution pattern, waist-to-hip ratio, left ventricular structure and function, sex differences, Mendelian randomization

## Abstract

**Background:**

Although there is an interaction between sex, body fat distribution, and cardiac structure and function, these relationships have not been fully elucidated yet. This study aims to reveal the causal relationship between genetic determinants of fat distribution pattern and function of the left ventricular structure in different sexes.

**Methods:**

Genetic variants for waist circumference, hip circumference, waist-to-hip ratio (WHR), and body mass index (BMI) were selected from genome-wide association studies conducted in European samples. The dataset for left ventricular (LV) parameters was obtained from over 35,000 European samples in the UK Biobank Cardiovascular Magnetic Resonance sub-study. Two-sample Mendelian randomization (MR) analysis was employed to explore causal relationships.

**Results:**

After adjusting for BMI, WHR shows a positive causal relationship with LV hypertrophy and a significant negative causal relationship with LV volume and diastolic function. In further subgroup analysis, we only found similar results in WHR among the female population (FWHR), while in the male population, there was no significant causal relationship between MWHR and LV hypertrophy and diastolic function. Additionally, in our MR analysis, no causal relationship was found between WHR and LVEF.

**Conclusions:**

This study indicates that the fat distribution pattern has unique effects on the structure and function of the LV, and these effects vary by sex. This study provides evidence for a causal relationship between fat distribution and LV structure and function across both sexes.

## Background

Heart failure (HF), as a terminal manifestation of various cardiovascular diseases, affects over 37 million people worldwide and incurs significant healthcare expenditure for prevention and treatment annually (1). Despite major advances in treatment, the 5-year mortality rate after a HF diagnosis remains approximately 50% (2). Effective prevention strategies are urgently needed to improve clinical outcomes of heart failure. Previous research has made significant efforts in early intervention for HF, with substantial evidence indicating that structural and functional alterations in the left ventricle (LV), as well as LV remodeling, are central pathological changes in heart failure (3, 4). Early stages of HF often present with asymptomatic LV structural and functional abnormalities (5). Therefore, identifying and managing risk factors that trigger left ventricular structural and functional damage are crucial for early prevention of HF.

The interrelationship between heart structure and function is closely intertwined. Any disruption or dysfunction in heart structure can have a significant impact on its ability to perform vital functions effectively. The CARDIA study, conducted over a span of more than 35 years, has revealed that early risk factors play a pivotal role in determining the outlook for cardiovascular disease. Among these factors, obesity stands out as the most influential factor affecting both the structure and function of the heart (6). As the weight of obese individuals increases, their total circulating blood volume simultaneously increases, imposing a greater burden on the heart and eventually resulting in detrimental cardiac structural and functional changes (7). In a study of 5,881 participants in the Framingham Heart Study, the risk of developing heart failure increased by 5% in males and 7% in females for each unit increase in BMI (8). Further research on obesity has revealed that visceral obesity might play a more important role in LV remodeling compared to general obesity (9). A recent scientific statement from the American Heart Association (AHA) demonstrates that abdominal obesity is recognized as an independent risk factor for cardiovascular disease (CVD), regardless of body mass index (BMI) (10). It is well-recognized that the evidence provided by observational studies is not entirely reliable. Not only does it fail to establish a causal relationship, but it may also be biased by too many confounding factors and reverse causality. Recently, Gao et al. utilized a Mendelian randomization approach to reveal the causal relationship between body fat distribution differences (waist-to-hip ratio) and detrimental ventricular structure and function (11). However, the study failed to further explore causal connections with other factors such as sex through subgroup analyses. Elucidating the interplay between sex, body fat distribution, and cardiac structure and function warrants further investigation.

Mendelian randomization (MR) uses genetic variation as an instrumental variable for exposure to explore whether risk factors and outcomes have a causal effect in observational data. MR relies on natural, randomly assorted genetic variations during meiosis, which is independent of traditional confounding factors, such as environmental, socioeconomic, and behavioral influences. This method can yield less biased results (12). In this study, waist circumference, hip circumference, and waist-to-hip ratio were used as exposure indicators to investigate their causal relationships with left heart structure and function. Furthermore, considering the influence of sex differences on fat distribution, we conducted separate MR analyses were conducted for males and females, to reveal the underlying associations between fat distribution and cardiac structure and function in different sexes.

## Method

### Study design

**Figure 1:**
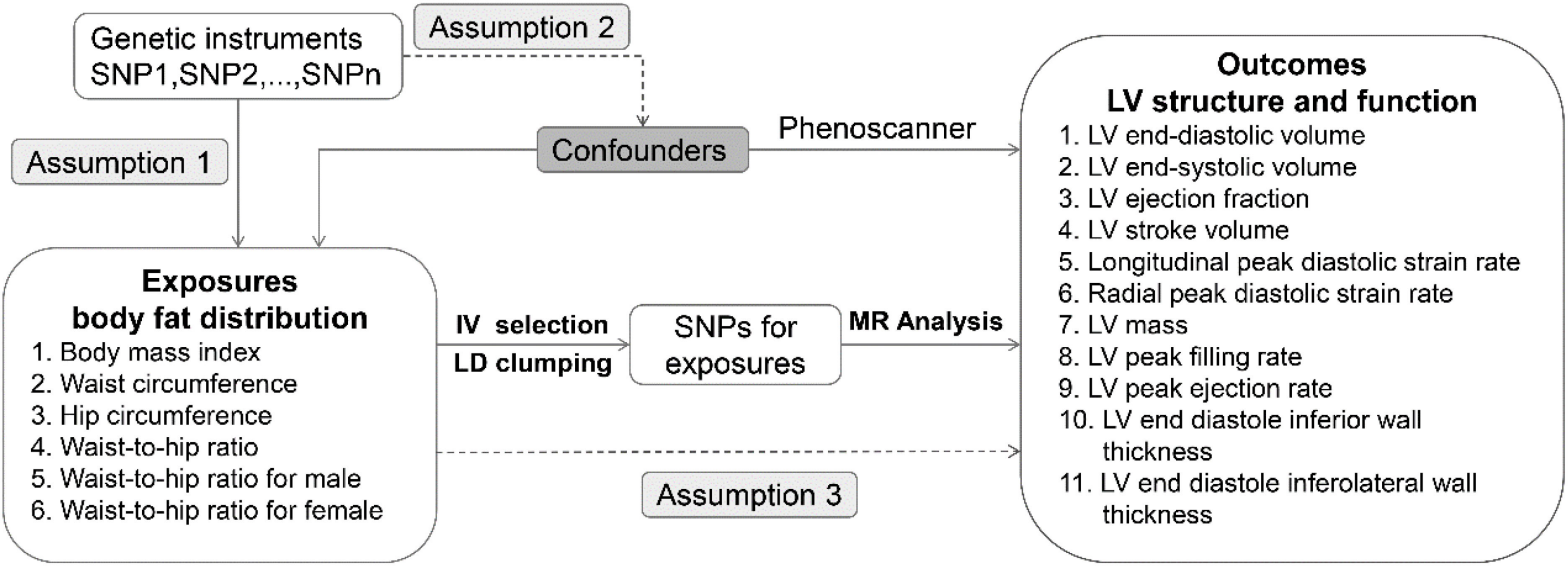
Study designs.

For this study, a two-sample MR design was employed. In selecting for genetic variants, MR relies on three fundamental assumptions (1): relevance assumption: the genetic variant is closely associated with the exposure of interest; (2) independence assumption: the genetic variant is not related to confounders that affect the exposure-outcome association; and (3) exclusion restriction: the genetic variant influences the outcome only through the exposure of interest. **Figure 1** shows the overview of the study design. All data are based on publicly available aggregated statistics from the genome-wide association studies (GWASs). For detailed information about these GWASs, refer to **Table 1**.

**Table 1:** Detailed clinical information of the GWASs used in present study.

Phenotype	PMID	Consortium	Sample size	Ancestry	Unit	Adjusted covariates
Exposures						
Body mass index	23563607	GIANT	16,068	European	SD (kg/m^2^)	
Waist circumference	25673412	GIANT	231,353	European	SD (cm)	Age, BMI
Hip circumference	25673412	GIANT	211,114	European	SD (cm)	Age, BMI
Waist-to-hip ratio	25673412	GIANT	210,082	European	SD	Age, BMI
Waist-to-hip ratio of male	25673412	GIANT	210,082	European	SD	Age, BMI
Waist-to-hip ratio of female	25673412	GIANT	210,082	European	SD	Age, BMI
Outcomes						
LV end-diastolic volume	32382064	UK Biobank	36,041	European	SD (ml)	
LV end-systolic volume	32382064	UK Biobank	36,041	European	SD (ml)	
LV ejection fraction	32382064	UK Biobank	36,041	European	SD (%)	
LV stroke volume	32382064	UK Biobank	36,041	European	SD (ml)	
Longitudinal peak diastolic strain rate	35479509	UK Biobank	39,559	European	SD (%/s)	
Radial peak diastolic strain rate	35479509	UK Biobank	39,559	European	SD (%/s)	
LV mass	36944631	UK Biobank	43,230	European	SD (g)	
LV peak filling rate	37126556	UK Biobank	32,526	European	SD (ml/s)	
LV peak ejection rate	37126556	UK Biobank	32,526	European	SD (ml/s)	
LV end diastole inferior wall thickness	38036550	UK Biobank	42,122	European	SD (mm)	
LV end diastole inferolateral wall thickness	38036550	UK Biobank	42,122	European	SD (mm)	

GWAS, genome-wide association studies; LV, left ventricular; GIANT, Genetic Investigation of Anthropometric Traits.

### Genetic instrument selection

Genetic variants for body fat distribution (waist and hip circumference-related traits) were selected from a large GWAS data, which included 224,459 individuals of European ancestry (13). In addition, genetic variants for BMI were selected from a GWAS data, which included 16,068 individuals of European ancestry (14). SNPs that reached the genome-wide significance level (*P* < 5×10^-8^) were selected. After linkage disequilibrium (LD) clumping at a threshold of r2 < 0.001 (clumping window: 10,000 kB), SNPs for waist circumference, hip circumference, waist-to-hip ratio, and BMI were identified respectively with exclusion of SNP associated with outcome and harmonization to exclude palindromic and incompatible SNPs using 1000 Genomes European Ancestry as a reference panel. F-statistics of all SNPs are over 10, indicating that the instruments have strong predictive potential. Details of all SNPs are listed in Supplemental Materials-SNP data.

### Data sources for outcomes

Cardiovascular magnetic resonance (CMR) is the gold standard technique for measuring biventricular volume, mass, wall thickness, and ejection fraction (EF) and is widely used to measure ventricular structure and function. In this study, LV structure and function parameters include eleven phenotypes: LV end-diastolic volume (LVEDV), LV end-systolic volume (LVESV), LV ejection fraction (LVEF), LV stroke volume (LVSV), longitudinal peak diastolic strain rate (LPDSR), radial peak diastolic strain rate (RPDSR), LV end diastole inferior wall thickness (LVEDIWT), LV end diastole inferolateral wall thickness (LVEDI-LWT), LV mass (LVM), LV peak filling rate (LVPFR) and LV peak ejection rate (LVPER). All pooled data for LV parameters were obtained from the UK Biobank cardiovascular magnetic resonance (CMR) study. The UK Biobank was approved by the North West Multi-Centre Research Ethics Committee and obtained the written informed consent of all participants (15).

### Statistical analysis

All analyses were performed in R software (version 4.1.1). “Mendelian randomization”, “TwoSampleMR” and “MRPresso” packages were used in the analysis. PhenoScanner (http://www.phenoscanner.medschl.cam.ac.uk), a platform that provides genotypic and phenotypic correlation information, was used to screen for the presence of these SNPs to correlate with potential risk factors. Potential confounders of the genome-wide significance of SNPs associated with these risk factors were then eliminated. In MR analysis, Inverse variance weighted (IVW), weighted median, MR-Egger, Simple mode, Weighted mode were used for MR estimations. The IVW method was performed using a random-effects meta-analysis model, which provides the most precise estimates though assuming that all SNPs are valid instruments (16). The weighted median method provides consistent estimates when the valid instrumental variance accounts for more than 50% of the weights. The MR-Egger regression produces estimates after accounting for cross-sectional pleiotropy, but the precision is low. The MR-PRESSO method identifies SNP outliers and provides the same results as IVW after removing the outliers. Cochran Q test, Egger-intercept test, and leave-one-out analysis were used for sensitivity analysis, which can be presented in scatter plots, funnel plot and forest plots. The two-sided *P* value of <0.05 was considered as statistically significant. To avoid the inflation of false-positive findings, we calculated the false-discovery rate (FDR) adjusted *P* values (*P*_FDR_) across the main analyses.

## Results

### Mendelian randomization analysis

**Figure 2:**
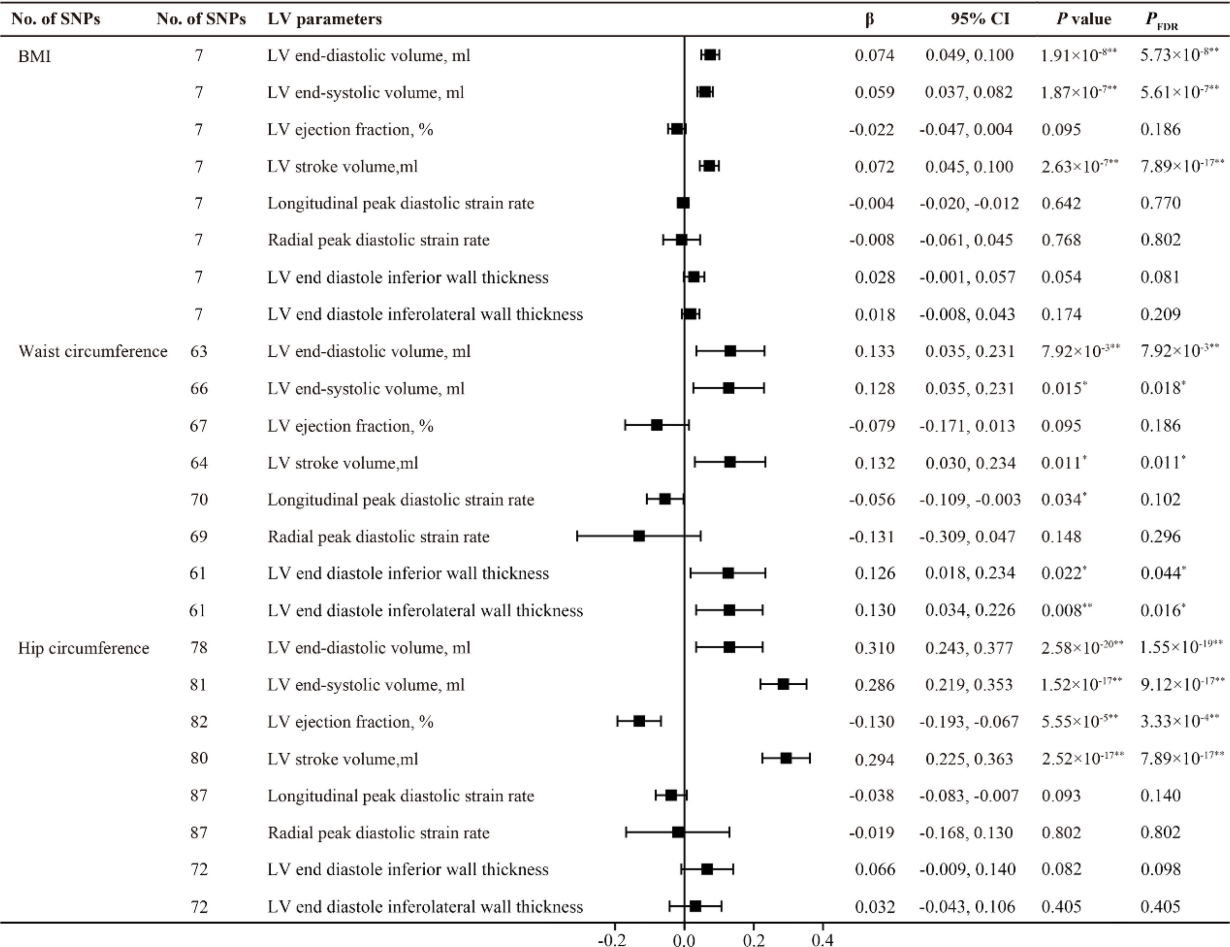
MR estimates of obesity traits on left ventricular parameters. Estimates are derived from IVW MR analyses. Data are presented as change in LV parameter per 1-SD increase in BMI, waist circumference and hip circumference. SNP, single nucleotide polymorphism; LV, left ventricular; BMI, body mass index. ^*^ *P*(*P*_FDR_) < 0.05, ^**^ *P*(*P*_FDR_) < 0.01.

**Figure 3:**
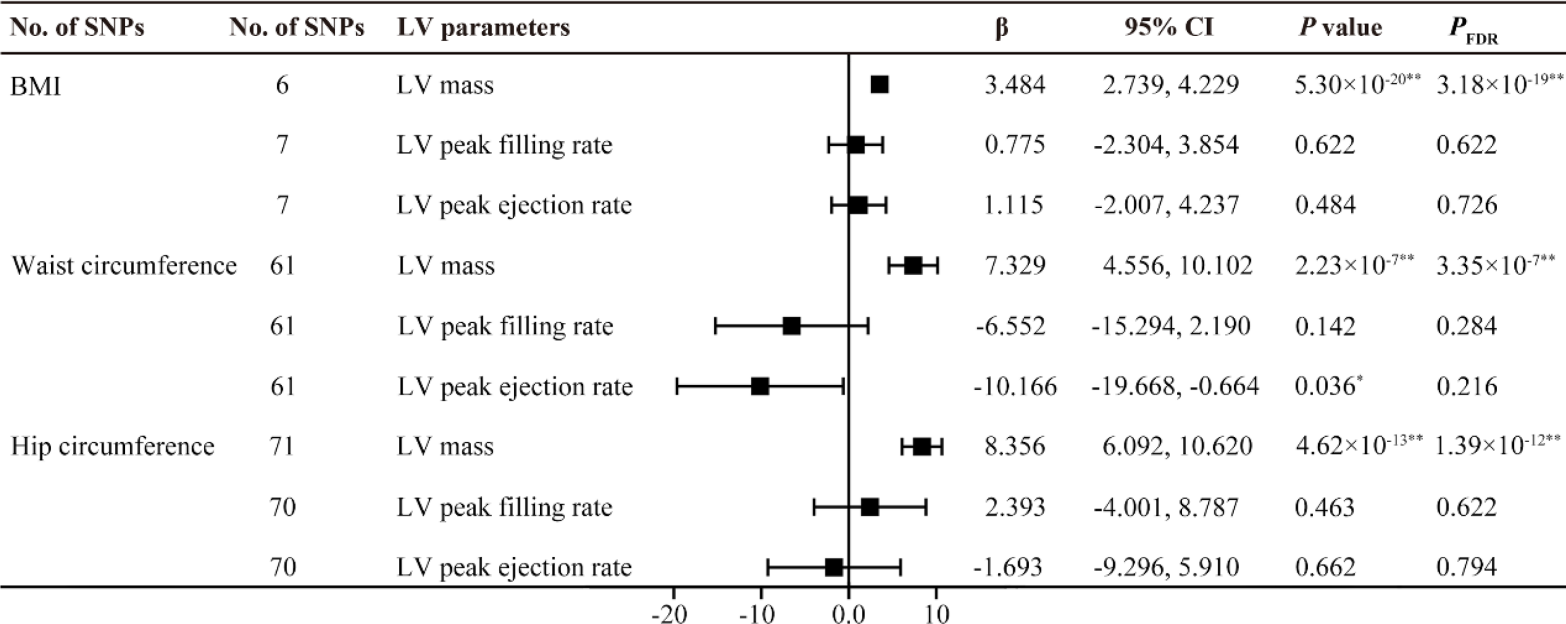
MR estimates of obesity traits on left ventricular parameters. Estimates are derived from IVW MR analyses. Data are presented as change in LV parameter per 1-SD increase in BMI, waist circumference and hip circumference. SNP, single nucleotide polymorphism; LV, left ventricular; BMI, body mass index. ^*^ *P*(*P*_FDR_) < 0.05, ^**^ *P*(*P*_FDR_) < 0.01.

All F statistics for the overall instruments were over 10, indicating a good strength of used genetic instruments. **Figures 2**, **3** shows the genetic association between obesity traits and LV parameters. In the primary IVW MR analyses, per 1-SD increase in genetic liability to BMI was positively correlated with LV end-diastolic volume (LVEDV) (β =0.074, 95% confidence interval (CI): 0.049-0.100, *P*_FDR_ =5.73×10^-8^), LV end-systolic volume (LVESV) (β =0.059, 95% CI: 0.037-0.082, *P*_FDR_ =5.61×10^-7^), LV stroke volume (LVSV) (β =0.072, 95% CI: 0.045-0.100, *P*_FDR_ =7.89×10^-17^), and LV mass (β =3.484, 95% CI: 2.739-4.229, *P*_FDR_ =3.18×10^-19^). In the analysis of this study, no significant causal relationship was found between BMI and LV ejection fraction (LVEF), LV longitudinal peak diastolic strain rate, radial peak diastolic strain rate (RPDSR), LV peak filling rate (LVPFR) and LV peak ejection rate (LVPER). Analysis of waist circumference (WC) and hip circumference (HC) yielded similar results.

However, the highlight here is that among BMI, WC, and HC, only WC was found to have a positive causal relationship with LV end diastole inferior wall thickness (LVEDIWT) (β =0.126, 95% CI: 0.018-0.234, *P*_FDR_ =0.044) and LV end diastole inferolateral wall thickness (LVEDI-LWT) (β =0.130, 95% CI: 0.034-0.226, *P*_FDR_ =0.016), suggesting that an increase in WC promotes left ventricular hypertrophy.

**Figure 4:**
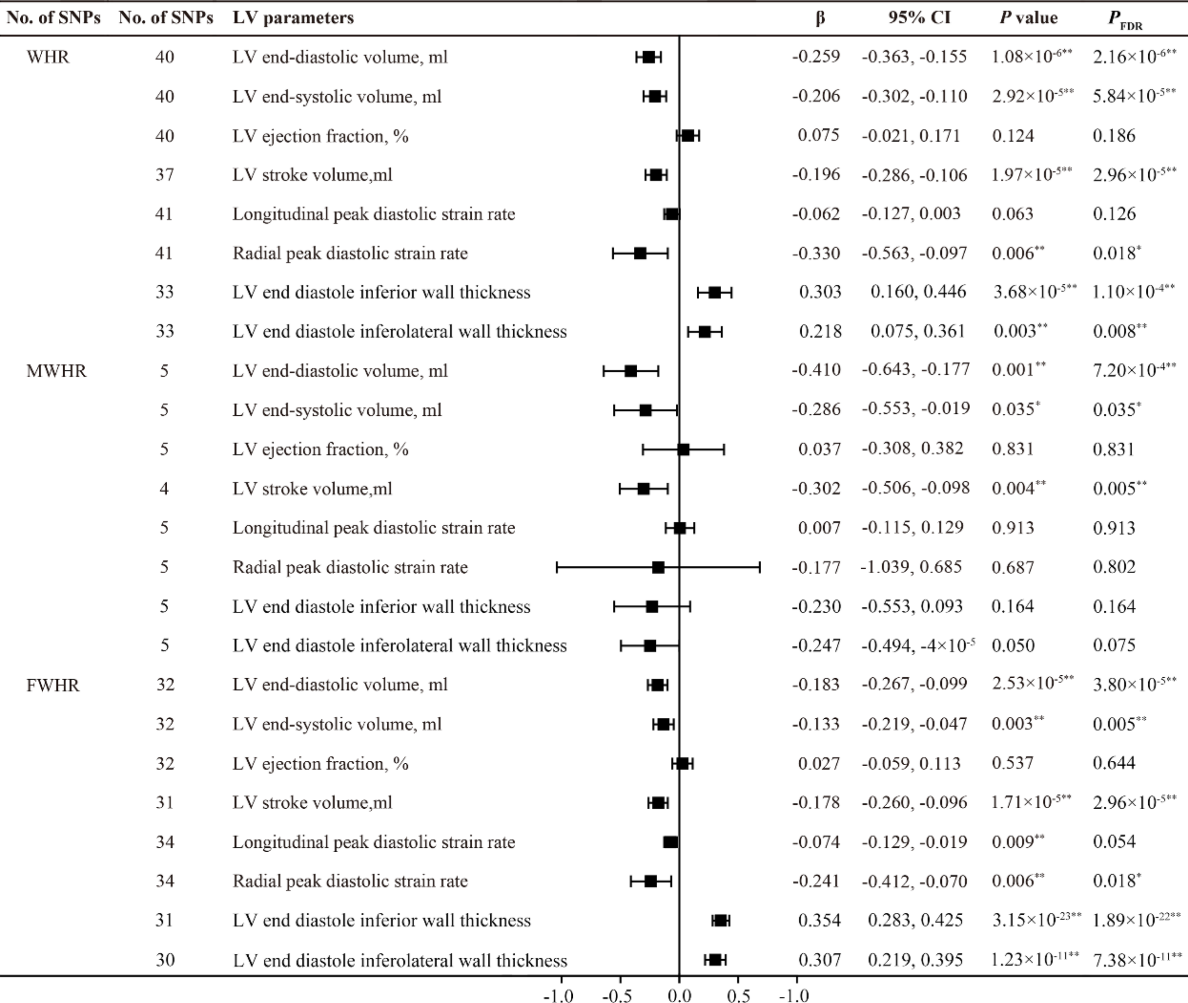
MR estimates of fat distribution pattern traits on left ventricular parameters. Estimates are derived from IVW MR analyses. Data are presented as change in LV parameter per 1-SD increase in WHR. SNP, single nucleotide polymorphism; LV, left ventricular; WHR, waist-to-hip ratio; MWHR, Waist-to-hip ratio of male; FWHR, waist-to-hip ratio of female. ^*^ *P*(*P*_FDR_) < 0.05, ^**^ *P*(*P*_FDR_) < 0.01.

**Figure 5:**
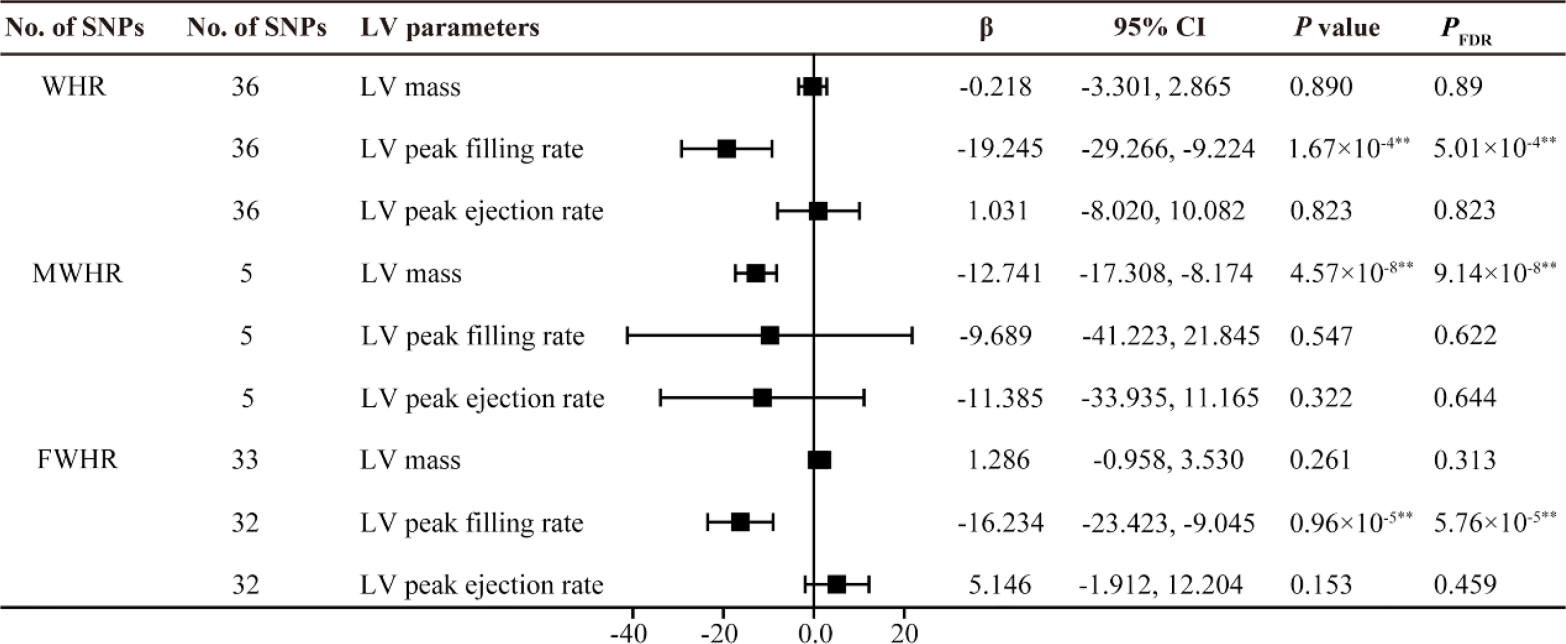
MR estimates of fat distribution pattern traits on left ventricular parameters. Estimates are derived from IVW MR analyses. Data are presented as change in LV parameter per 1-SD increase in WHR. SNP, single nucleotide polymorphism; LV, left ventricular; WHR, waist-to-hip ratio; MWHR, Waist-to-hip ratio of male; FWHR, waist-to-hip ratio of female. ^**^ *P*(*P*_FDR_) < 0.01.

After adjusting the BMI, the waist-to-hip ratio (WHR) showed a negative correlation with LVEDV, LVESV and LVSV. Similar results were also found in the subgroup analysis of WHR in males and females. Additionally, WHR exhibited a significant positive causal relationship with LVEDIWT (β =0.303, 95% CI 0.160-0.446, *P*_FDR_ =1.10×10^-4^) and LVEDI-LWT (β = 0.218, 95% CI 0.075-0.361, *P*_FDR_ =0.008), as well as a negative causal relationship with RPDSR (β = -0.330, 95% CI -0.563- -0.097, *P*_FDR_ =0.018). It is worth noting that we only obtained similar results in the female subgroup (FWHR); in males, no causal relationship was found between MWHR and left ventricular thickness and peak diastolic strain rate (**Figures 4**, **5**).

**Supplementary Tables S1**, **S2** shows the detailed information of additional MR methods for sensitivity analyses. For all analyses, the results from the weighted median and MR-Egger method supported a similar association. Due to lower statistical power, most estimates from these two methods were attenuated, but the direction of the results was consistent with the main IVW method.

### Heterogeneity and horizontal pleiotropy test

Cochran’s Q statistic was used to assess the heterogeneity of the instrument variable. The random-effects IVW model was used if the substantial heterogeneity was significant. A leave-one-out plots for IVW estimates was also used to confirm that the effects were not unduly influenced by outliers potentially representing pleiotropic pathways.

Horizontal pleiotropy refers to selected genetic instrument variables influences the outcome not via the exposure but other alternative pathways (17). In the results of this study, horizontal pleiotropy was detected between WC to LVEF and WC to LVSV (**Supplementary Table S3**). It is believed that this may be due to the fact that the majority of SNPs regulating WC, affect the outcome variables by regulating the lipid metabolism pathway, resulting in unavoidable horizontal pleiotropy. Despite repeated adjustments using leave-one-out analysis, this result could not be avoided. However, it is considered that this does not affect the main conclusions of this study. No heterogeneity and horizontal pleiotropy were observed in the remaining calculations. The leave-one-out analysis plots, forest plots, and funnel plots were shown in **Supplementary Figures S1**-**S18**.

## Discussion

In this two-sample MR study, the association of fat distribution patterns with the left ventricular structure and function was investigated, with genetic variants that proxy the effect identified from publicly available large-scale GWAS data. In this study, the causal relationship between body fat distribution patterns and LV structure and function was systematically assessed. The results of this study support the view that there is a causal relationship between increased waist-to-hip ratio (i.e., abdominal obesity) and worsening left ventricular diastolic function, which is robust in the female population.

In the past few decades, obesity has been a highly significant factor among numerous predictors of heart failure. Several population-based studies have indicated that systemic obesity and central obesity are major risk factors for HF development. Increased total blood volume in obese individuals directly increases cardiac preload. Additionally, excessive fat accumulation in the body leads to elevated basal metabolic rate, resulting in compensatory increase in cardiac contractility and subsequent alterations in cardiac structure, ultimately leading to heart failure (18–20). To address the issue of horizontal pleiotropy in MR analysis, a smaller sample size for the BMI dataset was ultimately selected. This study found a significant positive correlation between BMI, WC, HC, and the increase in left ventricular volume and mass. Additionally, a negative causal relationship between HC and LVEF is observed, while WC has a positive causal relationship with LV hypertrophy. Although the negative causal relationships between BMI, WC, and LVEF were not statistically significant, they exhibited similar trends (BMI-LVEF, *P*=0.095; WC-LVEF, *P*=0.095). These results are consistent with previous research, indicating that obesity contributes to left ventricular remodeling and potential cardiac dysfunction. Importantly, no causal association between BMI and cardiac diastolic parameters was found in this study. However, after adjusting for BMI, there is a negative causal relationship between WC and LPDSR, whereas no causal association was observed between HC and LV diastolic function. This result suggests that fat accumulation in specific body regions, rather than simple weight gain, plays a more significant role in driving impaired cardiac diastolic function.

Indeed, several studies have investigated the association between fat distribution and cardiac diastolic function. A follow-up study by Bryan Wilner et al. involving 1,262 participants also suggested that waist circumference (WC) is a better predictor of overall body fat than BMI, and found that abdominal obesity represented by WC is associated with concentric cardiac hypertrophy but unrelated to ejection fraction (EF) (21). Waist-to-hip ratio (WHR) is an ideal indicator for evaluating body fat distribution. In recent years, cardiac magnetic resonance imaging (CMR) has been used to assess LV diastolic function. Zou et al. conducted a study using left ventricular mass-to-end-diastolic volume ratio (LVMVR) as the outcome variable and employed MR to validate the negative causal relationship between waist-to-hip ratio (WHR) and cardiac diastolic function (11). However, in recent research, among various parameters measured by CMR, diastolic strain rate, particularly longitudinal diastolic strain rate, has been considered an ideal indicator for assessing ventricular diastolic function (22). LPDSR and RPDSR data from CMR examinations of nearly 40,000 participants in the UK Biobank were selected as representative outcome variables for cardiac diastolic function in the analysis of this study. The results suggest that the causal impact of WHR on LV structure and function is different from that of BMI, WC, and HC. The MR analysis shows that, after adjusting for BMI, WHR is positively causally related to left ventricular hypertrophy, negatively related to diastolic function, and not causally associated with LV systolic function (**Figures 4**, **5**). These results align with previous observational study findings, indicating the independent impact of abdominal fat distribution (central obesity) on LV structure and function. The results of this study mutually confirm the causal effect of abdominal obesity on impaired diastolic function. In addition, a significant impact of sex on fat distribution was observed, and further subgroup analysis specifically conducted to examine this effect.

Compared with males, females have differences in cardiac structure and function, including smaller LV cavity, lower stroke volume, more pronounced concentric remodeling of the left ventricle under pressure overload, higher left ventricular stiffness under the same pathological conditions, and more sensitive response to hypertension and obesity (23). Due to this difference, sex affects almost every aspect of HF, from epidemiology and risk factors to pathophysiology, treatment response and final outcome. The overall incidence risk of HF is similar between male and female, however males are prone to heart failure with reduced ejection fraction (HFrEF), while females are dominated by heart failure with preserved ejection fraction (HFpEF). About two-thirds of HFpEF patients are women, and women also tend to have more severe symptoms than men (24). Compared with HFrEF, obesity is a stronger risk factor for the development of HFpEF, and body mass index (BMI) increases the risk of HFpEF by 34% for every standard deviation increase (25). Sex hormones contribute to the differences in body fat distribution and related disease risks between males and females. Higher concentrations of estrogen are associated with fat storage in the hips and thighs, rather than in the visceral area (26). Studies on mice have shown that estrogen can prevent fat inflammation and fibrosis before obesity occurs (27). After menopause, there is a shift in fat deposition from subcutaneous to visceral fat in females, which is often accompanied by an increased risk of metabolic issues. Central obesity (visceral obesity) is more common in postmenopausal women, and this form of obesity is associated with deterioration of cardiac function, even in individuals with normal BMI (28). The mechanisms underlying estrogen-dependent body fat distribution are not fully understood, but they may involve increased mTOR signaling, suppression of autophagy, and inhibition of adipogenesis/lipid storage. Estrogen plays a crucial role in epigenetic regulation of adipogenic genes by interacting with enzymes that modify DNA methylation and histone tails through post-translational modifications (29). In the MR analysis of this study, a significant positive causal relationship was found between FWHR and LV hypertrophy and diastolic dysfunction, whereas no causal relationship was observed between MWHR and LV hypertrophy and diastolic parameters. These results indicate that body fat distribution has a unique impact on changes in LV hypertrophy and diastolic function in the female population.

Compared to subcutaneous adipose tissue, visceral adipose tissue (VAT) has a higher distribution of blood vessels and nerves, and contains more immune cells. It is also more active in secreting pro-inflammatory cytokines (30). Systemic microvascular inflammation caused by obesity is a core concept associated with the development of HFpEF. A recent prospective multinational study on HFpEF showed that 75% of HFpEF patients had coronary microvascular dysfunction, and it was associated with markers of HF (NT-proBNP) and cardiac dysfunction (ventricular/atrial strain) severity and systemic endothelial dysfunction (31). The susceptibility of the female population susceptibility to endothelial inflammation may reflect sex differences in immune responses. Male and female differ in the activation of adaptive and innate immune systems. Females generally exhibit more pro-inflammatory cytokines, activation of inflammatory T cells and overall higher inflammatory reactivity. This may be related to the role of estrogen in immune responses (32). Estrogen alters the phenotype of monocytes and macrophages, regulates the expression of pro-inflammatory cytokines and affects the expression of target genes (33). The link between acquired immune system activity and cardiovascular function has been confirmed in autoimmune diseases with severe cardiovascular manifestations (such as systemic sclerosis) (34, 35). Females have a much higher prevalence of autoimmune diseases, which demonstrates sex differences in immunity and inflammation (32), and autoimmune diseases themselves are associated with the development of diastolic dysfunction (34). This may explain why a negative causal relationship between abdominal obesity and diastolic function was observed only in the female population in this. So far, the underlying mechanisms behind sex differences in cardiac structure and function have not been fully revealed and require further basic research to elucidate. As far as the current results are concerned, simple weight loss could benefit male heart failure patients. However, for the female population, maintaining a good body proportion is particularly important in addition to controlling weight.

## Limitations

This study had certain limitations: (1) This study was confined to individuals of European descent, which limits the generalizability of the findings to other populations; (2) Pleiotropic effects were observed between WC-LVEF and WC-LVSV, likely because most SNPs that regulate BMI and WC influence outcome variables through modulation of lipid metabolism pathways, resulting in inevitable pleiotropy; (3) Limitations of the data source constrained the parameters available to assess diastolic function; inclusion of more clinically utilized measures such as E/e’ and E/A could have provided greater insight; (4) all exposure data from the individuals with preserved LVEF in UK Biobank led to healthy worker effect.

## Conclusions

In summary, this MR study supports the genetic causal relationship between WHR and adverse changes in LV structure and function. Notably, investigation in this study underscores the pronounced sex disparities in the nexus between body fat distribution and LV function. Specifically, the causal relationship between waist-to-hip ratio and left ventricular hypertrophy and diastolic dysfunction appears to be predominantly evident in the female population. To the best of our knowledge, this is the first evidence of sex differences in the causal relationship between body fat distribution and cardiac function. Further research on the underlying mechanisms of these differences may help shed light on the pathophysiology of HF.

## Data Availability

Publicly available datasets were analyzed in this study. This data can be found here: https://gwas.mrcieu.ac.uk/.
