# Supplementary material for: Effect of fat distribution on left ventricular structure and function in different sexes: a Mendelian randomization study
Source: Front Endocrinol (Lausanne). 2025 Feb 25;16:1355968. doi: 10.3389/fendo.2025.1355968 (PMC11893391; doi:10.3389/fendo.2025.1355968)
Supplement: Supplementary file 1 [file DataSheet1.pdf]

Table S1. Sensitivity MR analysis of the association between obesity parameters and left ventricular parameters.

	SNP	IVW		Weighted-median		MR-Egger		Simple mode		Weighted mode	
		β±SE	*P-value*	β±SE	*P-value*	β±SE	*P-value*	β±SE	*P-value*	β±SE	*P-value*
**Waist-to-hip ratio**											
LV end-diastolic volume	7	0.074±0.013	1.91×10^{-8}	0.059±0.016	3.27×10^{-4}	0.03±0.064	0.661	0.054±0.027	0.088	0.054±0.017	0.020
LV end-systolic volume	7	0.059±0.011	1.87×10^{-7}	0.051±0.015	7.33×10^{-4}	0.049±0.056	0.416	0.042±0.024	0.124	0.047±0.019	0.050
LV ejection fraction	7	-0.022±0.013	0.095	-0.024±0.016	0.136	-0.068±0.06	0.315	-0.023±0.023	0.355	-0.027±0.021	0.232
LV stroke volume	7	0.072±0.014	2.63×10^{-7}	0.053±0.016	1.15×10^{-3}	0.004±0.064	0.95	0.053±0.028	0.113	0.05±0.021	0.053
Longitudinal peak diastolic strain rate	7	-0.004±0.008	0.642	-0.005±0.01	0.594	-0.029±0.038	0.476	0.011±0.015	0.493	-0.01±0.013	0.476
Radial peak diastolic strain rate	7	-0.008±0.027	0.768	-0.029±0.036	0.426	0.055±0.127	0.68	-0.053±0.06	0.409	-0.046±0.045	0.352
LV mass	6	3.484±0.380	5.30×10^{-20}	3.602±0.547	4.6E-11	3.980±2.280	0.156	3.962±0.860	0.006	3.962±0.873	0.006
LV peak filling rate	7	0.775±1.571	0.622	2.973±1.989	0.135	-8.087±6.905	0.294	3.954±3.172	0.259	3.898±4.001	0.368
LV peak ejection rate	7	1.115±1.593	0.484	0.372±1.840	0.84	0.116±8.109	0.989	0.461±2.728	0.871	-0.729±2.381	0.770
LV end diastole inferior wall thickness	7	0.028±0.015	0.054	0.037±0.018	0.035	0.048±0.075	0.544	0.054±0.029	0.111	0.042±0.024	0.123
LV end diastole inferolateral wall thickness	7	0.018±0.013	0.174	0.006±0.017	0.698	-0.026±0.060	0.685	0.020±0.026	0.469	0.010±0.022	0.672
**Waist-to-hip ratio for male**											
LV end-diastolic volume	63	0.133±0.050	7.92×10^{-3}	0.251±0.053	2.05×10^{-6}	0.343±0.221	0.127	0.327±0.117	6.76×10^{-3}	0.332±0.120	7.31×10^{-3}
LV end-systolic volume	66	0.128±0.052	0.015	0.208±0.054	0.0001	0.574±0.220	0.011	0.253±0.134	0.064	0.253±0.126	0.048
LV ejection fraction	67	-0.079±0.047	0.095	-0.094±0.060	0.115	-0.536±0.195	0.008	-0.062±0.147	0.674	-0.078±0.146	0.593
LV stroke volume	64	0.132±0.052	0.011	0.170±0.056	0.002	0.237±0.221	0.288	0.259±0.131	0.053	0.242±0.107	0.027
Longitudinal peak diastolic strain rate	70	-0.056±0.027	0.034	-0.037±0.033	0.259	-0.084±0.116	0.470	-0.024±0.083	0.774	-0.039±0.072	0.59
Radial peak diastolic strain rate	69	-0.131±0.091	0.148	-0.038±0.111	0.736	-0.321±0.398	0.422	0.039±0.253	0.877	0.039±0.238	0.87
LV mass	61	7.329±1.415	2.23×10^{-7}	7.954±1.414	1.85E-08	12.487±6.013	0.0422	8.918±3.071	0.005	8.918±2.826	0.003
LV peak filling rate	61	-6.552±4.460	0.142	-1.365±6.038	0.821	30.773±20.677	0.142	15.341±16.590	0.359	18.854±16.732	0.264
LV peak ejection rate	61	-10.166±4.848	0.036	-13.214±6.118	0.031	-15.730±4.460	0.499	-18.208±14.228	0.206	-17.067±13.455	0.21
LV end diastole inferior wall thickness	61	0.126±0.055	0.022	0.182±0.056	0.001	-0.011±0.232	0.963	0.226±0.120	0.065	0.209±0.108	0.057

LV end diastole inferolateral wall thickness	61	0.130±0.049	0.008	0.163±0.055	0.003	0.130±0.209	0.537	0.171±0.123	0.170	0.182±0.112	0.109
**Waist-to-hip ratio for female**											
LV end-diastolic volume	78	0.310±0.034	2.58×10^{-20}	0.298±0.042	1.80×10^{-12}	0.194±0.131	0.142	0.382±0.125	3.04×10^{-3}	0.178±0.095	0.063
LV end-systolic volume	81	0.286±0.034	1.52×10^{-17}	0.258±0.042	6.04×10^{-10}	0.289±0.127	0.025	0.241±0.112	0.035	0.161±0.090	0.078
LV ejection fraction	82	-0.130±0.032	5.55×10^{-5}	-0.103±0.044	0.018	-0.128±0.122	0.297	-0.036±0.108	0.741	-0.025±0.087	0.771
LV stroke volume	80	0.294±0.035	2.52×10^{-17}	0.258±0.045	1.10×10^{-8}	0.214±0.132	0.109	0.377±0.129	0.004	0.131±0.093	0.162
Longitudinal peak diastolic strain rate	87	-0.038±0.023	0.093	-0.048±0.029	0.103	-0.025±0.088	0.775	-0.084±0.083	0.316	-0.072±0.080	0.366
Radial peak diastolic strain rate	87	-0.019±0.076	0.802	-0.001±0.094	0.998	-0.099±0.292	0.736	0.040±0.253	0.874	0.019±0.201	0.923
LV mass	71	8.356±1.155	4.62×10^{-13}	7.950±1.271	3.968E-10	10.352±4.343	0.020	10.682±3.070	4.67×10^{-4}	8.430±2.295	4.64×10^{-4}
LV peak filling rate	70	2.393±3.262	0.463	-3.053±4.840	0.528	7.442±12.493	0.553	-4.576±11.039	0.680	-6.442±11.097	0.563
LV peak ejection rate	70	-1.693±3.879	0.662	-5.865±5.019	0.243	-12.226±14.816	0.412	-4.314±12.384	0.729	-3.167±11.368	0.781
LV end diastole inferior wall thickness	72	0.066±0.038	0.082	0.111±0.046	0.015	0.131±0.142	0.356	0.203±0.093	0.033	0.199±0.084	0.021
LV end diastole inferolateral wall thickness	72	0.032±0.038	0.405	0.107±0.046	0.021	0.263±0.139	0.062	0.138±0.122	0.262	0.192±0.109	0.081

LV, left ventricular; MR, Mendelian randomization; IVW, inverse-variance weighted; SE, standard error.

Table S2. Sensitivity MR analysis of the association between body fat distribution and left ventricular parameters.

	SNP	IVW		Weighted-median		MR-Egger		Simple mode		Weighted mode	
		β±SE	*P-value*	β±SE	*P-value*	β±SE	*P-value*	β±SE	*P-value*	β±SE	*P-value*
**Waist-to-hip ratio**											
LV end-diastolic volume	40	-0.259±0.053	1.08×10^{-6}	-0.198±0.057	5.18×10^{-4}	-0.171±0.261	0.515	-0.229±0.122	0.067	-0.203±0.100	0.048
LV end-systolic volume	40	-0.206±0.049	2.92×10^{-5}	-0.124±0.061	0.041	-0.080±0.241	0.742	-0.110±0.139	0.435	-0.106±0.113	0.356
LV ejection fraction	40	0.075±0.049	0.124	-0.038±0.063	0.543	-0.058±0.239	0.809	-0.088±0.135	0.519	-0.071±0.109	0.517
LV stroke volume	37	-0.196±0.046	1.97×10^{-5}	-0.212±0.062	6.51×10^{-4}	0.088±0.250	0.727	-0.242±0.134	0.079	-0.226±0.111	0.050
Longitudinal peak diastolic strain rate	41	-0.062±0.033	0.063	-0.055±0.042	0.197	-0.038±0.165	0.820	-0.029±0.083	0.729	-0.049±0.067	0.470
Radial peak diastolic strain rate	41	-0.330±0.119	0.006	-0.280±0.132	0.033	-0.164±0.586	0.781	-0.333±0.301	0.274	-0.279±0.231	0.234
LV mass	36	-0.218±1.573	0.890	1.430±1.669	0.392	5.734±7.563	0.454	4.506±3.284	0.179	4.506±3.525	0.210
LV peak filling rate	36	-19.245±5.113	1.67×10^{-4}	-22.962±6.851	8.03×10^{-4}	-26.874±24.766	0.285	-24.741±14.566	0.098	-27.420±15.169	0.079
LV peak ejection rate	36	1.031±4.618	0.823	2.623±6.593	0.731	-11.342±22.079	0.611	-1.461±14.670	0.921	7.318±14.119	0.607
LV end diastole inferior wall thickness	33	0.303±0.073	3.68×10^{-5}	0.405±0.073	2.89×10^{-8}	0.905±0.338	0.012	0.401±0.152	0.013	0.417±0.115	9.39×10^{-4}
LV end diastole inferolateral wall thickness	33	0.218±0.073	0.003	0.270±0.073	2.10×10^{-4}	0.757±0.337	0.032	0.386±0.137	0.008	0.326±0.112	0.007
**Waist-to-hip ratio for male**											
LV end-diastolic volume	5	-0.410±0.119	0.001	-0.278±0.132	0.035	-3.844±2.280	0.190	-0.210±0.190	0.331	-0.208±0.168	0.283
LV end-systolic volume	5	-0.286±0.136	0.035	-0.157±0.129	0.225	-0.814±0.3.441	0.828	-0.061±0.187	0.761	-0.061±0.177	0.747
LV ejection fraction	5	0.037±0.176	0.831	-0.124±0.134	0.354	-3.431±3.978	0.452	-0.153±0.154	0.377	-0.148±0.136	0.338
LV stroke volume	4	-0.302±0.104	0.004	-0.306±0.120	0.011	-2.221±4.210	0.651	-0.340±0.157	0.119	-0.339±0.151	0.111
Longitudinal peak diastolic strain rate	5	0.007±0.062	0.913	0.060±0.080	0.452	-1.841±1.357	0.268	0.071±0.111	0.557	0.071±0.114	0.567
Radial peak diastolic strain rate	5	-0.177±0.440	0.687	-0.185±0.353	0.600	-17.172±5.263	0.047	-0.197±0.553	0.740	-0.144±0.594	0.820
LV mass	5	-12.741±2.330	4.57×10^{-8}	-12.719±3.323	1.29×10^{-4}	-52.972±52.206	0.377	-12.531±4.298	0.043	-12.489±4.491	0.065
LV peak filling rate	5	-9.689±16.089	0.547	-16.052±15.568	0.302	-133.357±401.213	0.761	-30.407±23.612	0.267	-27.720±21.267	0.261
LV peak ejection rate	5	-11.385±11.505	0.322	-13.658±13.652	0.317	480.026±222.540	0.120	-18.979±20.054	0.398	-17.353±21.833	0.471
LV end diastole inferior wall thickness	5	-0.230±0.165	0.164	-0.004±0.151	0.978	3.566±3.562	0.390	0.022±0.203	0.918	0.030±0.189	0.882

LV end diastole inferolateral wall thickness	5	-0.247±0.126	0.050	-0.292±0.138	0.035	-0.826±3.184	0.812	-0.368±0.188	0.122	-0.350±0.185	0.132
**Waist-to-hip ratio for female**											
LV end-diastolic volume	32	-0.183±0.043	2.53×10^{-5}	-0.171±0.047	2.86×10^{-4}	-0.111±0.173	0.529	-0.224±0.102	3.52×10^{-2}	-0.159±0.097	0.011
LV end-systolic volume	32	-0.133±0.044	0.003	-0.096±0.050	0.056	-0.012±0.176	0.944	-0.167±0.100	0.106	-0.118±0.079	0.146
LV ejection fraction	32	0.027±0.044	0.537	0.052±0.055	0.346	-0.117±0.174	0.505	-0.047±0.096	0.630	0.029±0.077	0.713
LV stroke volume, ml	31	-0.178±0.042	1.71×10^{-5}	-0.181±0.052	5.14×10^{-4}	-0.055±0.171	0.751	-0.188±0.109	0.094	-0.166±0.115	0.159
Longitudinal peak diastolic strain rate	34	-0.074±0.028	0.009	-0.070±0.034	0.039	-0.148±0.111	0.191	-0.088±0.072	0.234	-0.081±0.051	0.120
Radial peak diastolic strain rate	34	-0.241±0.087	0.006	-0.183±0.113	0.104	-0.208±0.342	0.574	-0.295±0.265	0.275	-0.104±0.187	0.583
LV mass	33	1.286±1.145	0.261	1.903±1.316	0.148	4.213±4.410	0.347	3.077±2.671	0.258	3.184±2.266	0.170
LV peak filling rate	32	-16.234±3.668	9.60×10^{-6}	-15.545±5.092	2.27×10^{-3}	-32.713±14.059	0.027	-27.885±10.158	9.97×10^{-3}	-16.321±8.656	0.069
LV peak ejection rate	32	5.146±3.601	0.153	6.871±5.240	0.190	-3.193±13.804	0.819	2.792±10.302	0.788	4.563±10.288	0.660
LV end diastole inferior wall thickness	31	0.354±0.036	3.15×10^{-23}	0.367±0.055	2.06×10^{-11}	0.364±0.137	0.0129	0.466±0.110	1.96×10^{-4}	0.434±0.090	4.04×10^{-5}
LV end diastole inferolateral wall thickness	30	0.307±0.045	1.23×10^{-11}	0.259±0.055	2.88×10^{-6}	0.326±0.180	0.081	0.243±0.117	0.046	0.250±0.086	0.007

LV, left ventricular; MR, Mendelian randomization; IVW, inverse-variance weighted; SE, standard error.

Table S3. **Heterogeneity and horizontal pleiotropy test of the associations between body fat distribution and left ventricular parameters.**

	Weak instruments	IVW		MR-Egger				
	**Mean F-value**	**Cochran's Q**	***P-value***	**Cochran's Q**	***P-value***	**Intercept**	**SE**	***P-value* for intercept**
**Body mass index**								
LV end-diastolic volume	52.630	8.624	0.196	7.840	0.165	0.011	0.016	0.511
LV end-systolic volume	52.630	5.543	0.476	5.505	0.357	0.002	0.013	0.860
LV ejection fraction	52.630	1.910	0.928	1.307	0.934	0.011	0.015	0.473
LV stroke volume	52.630	8.418	0.209	6.824	0.234	0.017	0.016	0.329
Longitudinal peak diastolic strain rate	52.630	3.394	0.758	2.922	0.712	0.006	0.009	0.523
Radial peak diastolic strain rate	52.630	4.756	0.575	4.493	0.481	-0.016	0.031	0.630
LV and diastole inferior wall thickness	52.630	7.787	0.254	7.671	0.175	-0.005	0.018	0.794
LV end diastole inferolateral wall thickness	52.627	4.393	0.624	3.845	0.572	0.0110	0.015	0.493
LV mass	37.705	5.262	0.385	5.198	0.268	-0.112	0.505	0.836
LV peak filling rate	52.627	8.163	0.226	0.068	0.300	2.191	1.668	0.246
LV peak ejection rate	52.627	8.711	0.191	8.683	0.122	0.247	1.958	0.905
**Waist circumference**								
LV end-diastolic volume	47.560	154.000	8.69×10^{-10}	151.650	1.12×10^{-9}	-0.006	0.006	0.335
LV end-systolic volume	47.780	175.350	4.44×10^{-12}	164.270	9.23×10^{-11}	-0.006	0.006	0.042*
LV ejection fraction	47.240	112.150	3.00×10^{-12}	102.940	0.002	0.012	0.005	0.019*
LV stroke volume	47.560	149.220	5.92×10^{-9}	148.650	4.47×10^{-9}	-0.003	0.006	0.627
Longitudinal peak diastolic strain rate	47.240	98.310	0.012	98.220	9.60×10^{-3}	0.001	0.003	0.804
Radial peak diastolic strain rate	47.520	99.920	0.007	99.560	0.006	0.005	0.010	0.626
LV and diastole inferior wall thickness	47.812	127.113	9.95×10^{-7}	126.232	8.32×10^{-7}	0.004	0.006	0.546
LV end diastole inferolateral wall thickness	47.812	102.374	5.40×10^{-4}	102.374	4.00×10^{-4}	1.67×10^{-5}	0.005	0.998

LV mass	47.950	152.080	6.01×10^{-10}	150.100	6.96×10^{-10}	-0.137	0.156	0.381
LV peak filling rate	46.670	75.166	0.09	71.057	0.135	-0.965	0.523	0.070
LV peak ejection rate	46.670	92.111	0.005	92.017	0.004	0.144	0.584	0.806
**Hip circumference**								
LV end-diastolic volume	52.970	136.260	3.68×10^{-5}	134.760	3.84×10^{-5}	0.004	0.004	0.361
LV end-systolic volume	53.630	140.660	3.32×10^{-5}	140.660	2.44×10^{-5}	-101.200	0.004	0.980
LV ejection fraction	53.600	103.190	0.049	103.190	0.042	-68.000	0.004	0.986
LV stroke volume	52.840	133.870	1.15×10^{-4}	133.210	9.97×10^{-5}	0.002	0.004	0.535
Longitudinal peak diastolic strain rate	53.630	147.540	4.12×10^{-5}	147.500	3.11×10^{-5}	-4.00×10^{-4}	0.003	0.877
Radial peak diastolic strain rate	53.630	144.170	8.661×10^{-5}	144.040	6.73×10^{-5}	0.002	0.009	0.778
LV and diastole inferior wall thickness	56.713	115.342	0.001	114.973	5.70×10^{-4}	-0.002	0.004	0.637
LV end diastole inferolateral wall thickness	56.713	115.384	0.001	110.640	0.001	-0.007	0.004	0.088
LV mass	55.600	180.638	1.00×10^{-11}	180.045	7.41×10^{-12}	-0.062	0.131	0.635
LV peak filling rate	54.524	71.042	0.410	70.859	0.383	-0.155	0.370	0.677
LV peak ejection rate	54.524	104.234	0.004	103.408	0.004	0.324	0.439	0.464
**Waist-to-hip ratio**								
LV end-diastolic volume	52.560	81.040	8.96×10^{-5}	80.780	6.42×10^{-5}	-0.003	0.007	0.733
LV end-systolic volume	52.560	65.620	0.005	65.130	0.004	-0.004	0.007	0.597
LV ejection fraction	52.560	49.610	0.119	49.190	0.106	0.004	0.007	0.572
LV stroke volume	51.650	43.780	0.175	42.170	0.189	-0.008	0.007	0.256
Longitudinal peak diastolic strain rate	52.920	61.980	0.014	61.940	0.011	0.001	0.005	0.880
Radial peak diastolic strain rate	52.920	69.730	0.002	69.580	0.002	-0.005	0.017	0.773
LV and diastole inferior wall thickness	53.665	77.440	1.23×10^{-5}	69.966	7.72×10^{-5}	-0.018	0.010	0.078
LV end diastole inferolateral wall thickness	53.670	75.605	2.18×10^{-5}	69.604	8.61×10^{-5}	-0.157	0.010	0.112
LV mass	53.847	75.841	7.67×10^{-5}	74.423	7.59×10^{-5}	-0.173	0.215	0.427
LV peak filling rate	53.847	41.363	0.213	41.243	0.183	0.222	0.704	0.755

LV peak ejection rate	53.847	31.280	0.648	30.952	0.618	0.360	0.628	0.570
**Waist-to-hip ratio for male**								
LV end-diastolic volume	57.290	8.000	0.091	4.550	0.208	0.115	0.076	0.229
LV end-systolic volume	57.290	9.800	0.043	9.740	0.021	0.018	0.115	0.888
LV ejection fraction	57.290	12.610	0.013	10.060	0.018	0.116	0.133	0.447
LV stroke volume	57.620	0.240	0.970	0.0350	0.983	0.063	0.138	0.693
Longitudinal peak diastolic strain rate	57.290	2.970	0.562	1.120	0.773	0.062	0.045	0.266
Radial peak diastolic strain rate	57.290	17.920	0.001	4.000	0.262	0.569	0.176	0.048*
LV and diastole inferior wall thickness	37.246	11.203	0.024	8.122	0.044	-0.127	0.119	0.364
LV end diastole inferolateral wall thickness	37.246	6.557	0.161	6.485	0.090	0.019	0.107	0.867
LV mass	37.246	3.362	0.499	2.744	0.433	1.347	1.713	0.489
LV peak filling rate	37.246	9.700	0.046	9.402	0.024	4.145	13.433	0.778
LV peak ejection rate	37.246	5.144	0.273	0.258	0.968	-16.45	7.451	0.114
**Waist-to-hip ratio for female**								
LV end-diastolic volume	37.250	61.730	0.001	61.350	0.001	-0.003	0.007	0.670
LV end-systolic volume	37.250	60.390	0.001	59.400	0.001	-0.005	0.007	0.483
LV ejection fraction	37.250	45.840	0.042	44.740	0.041	0.006	0.007	0.397
LV stroke volume	35.470	27.690	0.228	27.160	0.205	0.005	0.008	0.518
Longitudinal peak diastolic strain rate	37.250	57.160	0.006	56.330	0.005	0.003	0.004	0.495
Radial peak diastolic strain rate	37.250	47.680	0.0470	47.670	0.037	-0.001	0.013	0.921
LV and diastole inferior wall thickness	59.114	29.760	0.478	29.753	0.426	-0.0004	0.005	0.936
LV end diastole inferolateral wall thickness	57.279	43.632	0.040	43.613	0.03	-0.0008	0.007	0.913
LV mass	57.988	61.807	0.001	60.878	0.001	-0.116	0.168	0.497
LV peak filling rate	58.734	20.854	0.916	19.380	0.932	0.648	0.534	0.234
LV peak ejection rate	58.734	25.478	0.746	25.086	0.721	0.328	0.524	0.536

LV, left ventricular; IVW, inverse-variance weighted; MR, Mendelian randomization. *$P < 0.05$.

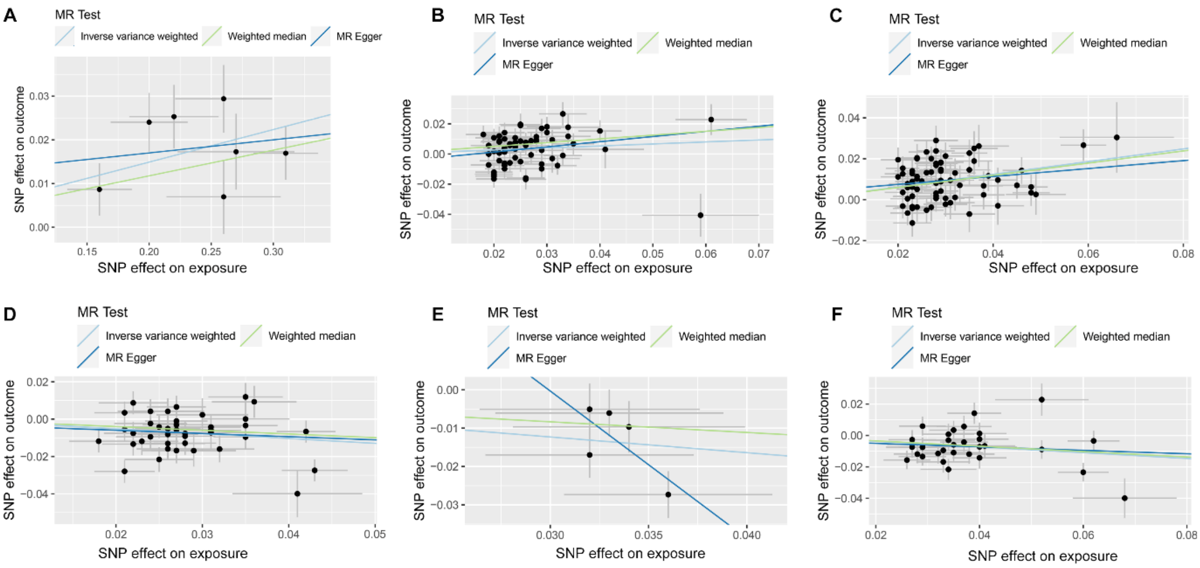

Figure S1. Scatterplots of the causal estimates of body fat distribution and left ventricular end-diastolic volume. Scatterplots of SNP effects on BMI (A), Waist circumference (B), Hip circumference (C), Waist-to-hip ratio (D), Waist-to-hip ratio for male (E), Waist-to-hip ratio for female (F) versus their effects on LV end-diastolic volume, with the slope of each line corresponding to the estimated MR effect of inverse variance-weighted, Weighted median, and MR-Egger, respectively. Error bars indicate 95% CIs. SNPs, single nucleotide polymorphisms; LV, left ventricular; BMI, Body mass index.

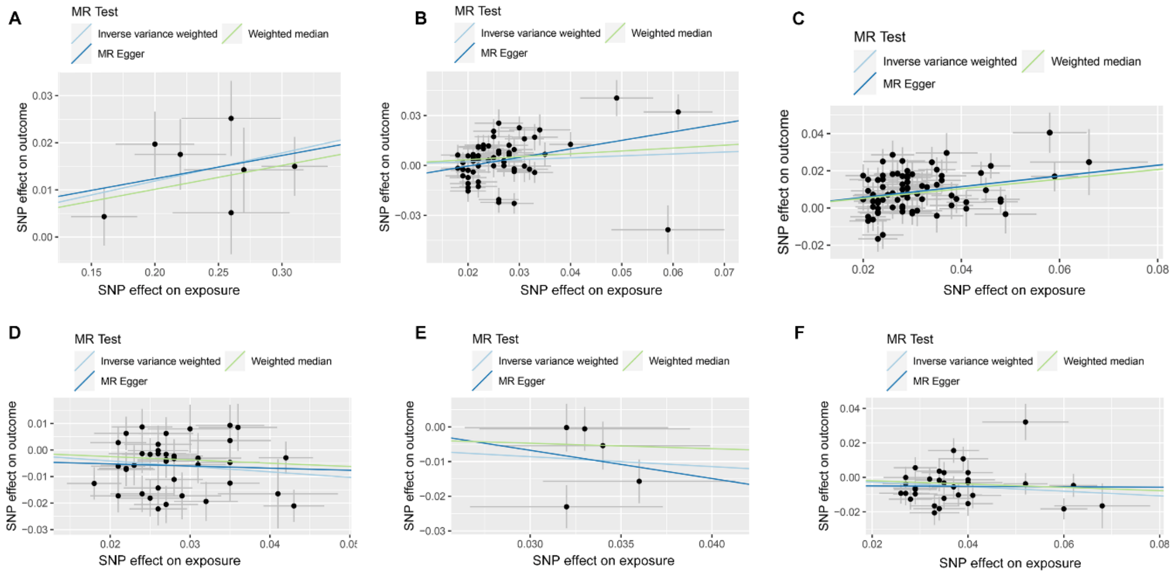

Figure S2. Scatterplots of the causal estimates of body fat distribution and left ventricular end-systolic volume. Scatterplots of SNP effects on BMI (A), Waist circumference (B), Hip circumference (C), Waist-to-hip ratio (D), Waist-to-hip ratio for male (E), Waist-to-hip ratio for female (F) versus their effects on LV end-systolic volume, with the slope of each line corresponding to the estimated MR effect of inverse variance-weighted, Weighted median, and MR-Egger, respectively. Error bars indicate 95% CIs. SNPs, single nucleotide polymorphisms; LV, left ventricular; BMI, Body mass index.

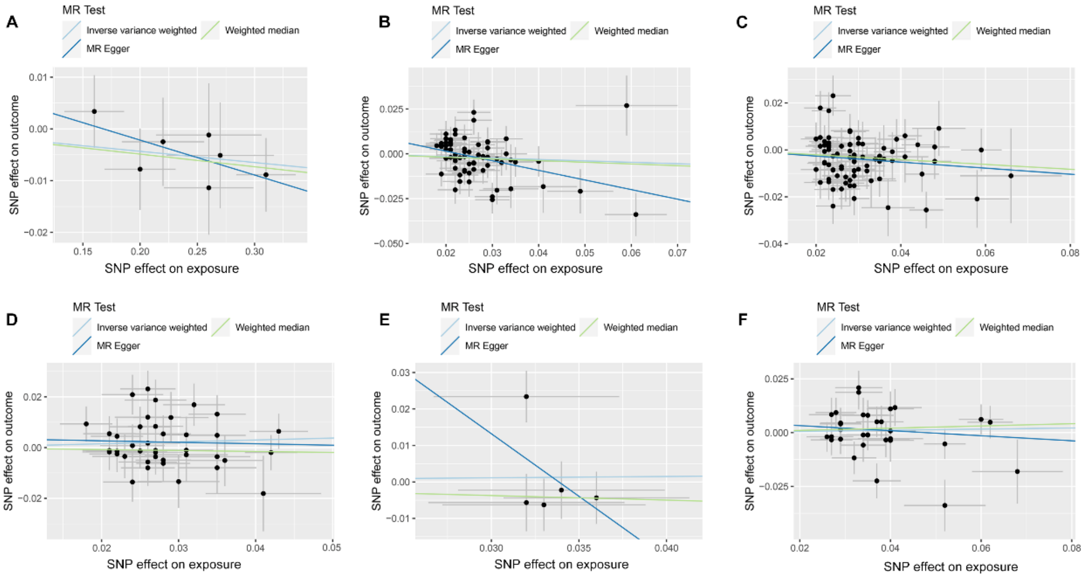

Figure S3. Scatterplots of the causal estimates of body fat distribution and left ventricular ejection fraction. Scatterplots of SNP effects on BMI (A), Waist circumference (B), Hip circumference (C), Waist-to-hip ratio (D), Waist-to-hip ratio for male (E), Waist-to-hip ratio for female (F) versus their effects on LV ejection fraction, with the slope of each line corresponding to the estimated MR effect of inverse variance-weighted, Weighted median, and MR-Egger, respectively. Error bars indicate 95% CIs. SNPs, single nucleotide polymorphisms; LV, left ventricular; BMI, Body mass index.

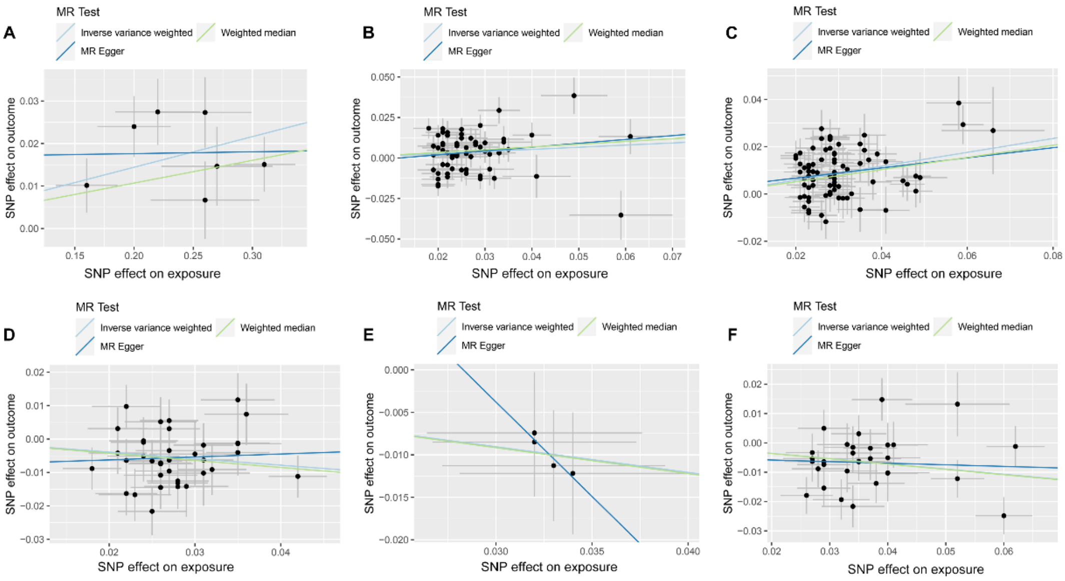

Figure S4. Scatterplots of the causal estimates of body fat distribution and left ventricular stroke volume. Scatterplots of SNP effects on BMI (A), Waist circumference (B), Hip circumference (C), Waist-to-hip ratio (D), Waist-to-hip ratio for male (E), Waist-to-hip ratio for female (F) versus their effects on LV stroke volume, with the slope of each line corresponding to the estimated MR effect of inverse variance-weighted, Weighted median, and MR-Egger, respectively. Error bars indicate 95% CIs. SNPs, single nucleotide polymorphisms; LV, left ventricular; BMI, Body mass index.

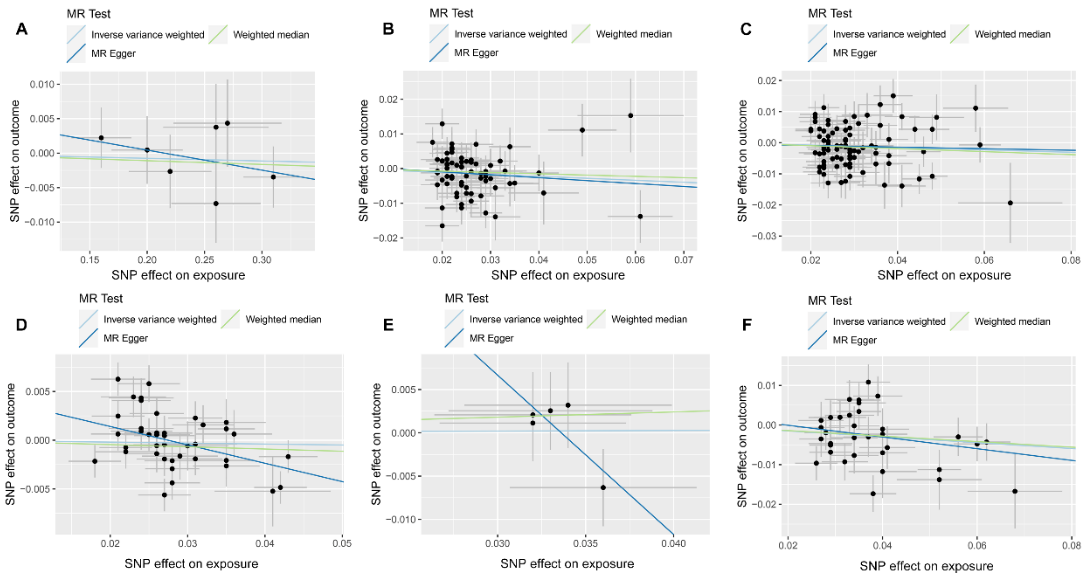

Figure S5. Scatterplots of the causal estimates of body fat distribution and Longitudinal peak diastolic strain rate. Scatterplots of SNP effects on BMI (A), Waist circumference (B), Hip circumference (C), Waist-to-hip ratio (D), Waist-to-hip ratio for male (E), Waist-to-hip ratio for female (F) versus their effects on Longitudinal peak diastolic strain rate, with the slope of each line corresponding to the estimated MR effect of inverse variance-weighted, Weighted median, and MR-Egger, respectively. Error bars indicate 95% CIs. SNPs, single nucleotide polymorphisms; LV, left ventricular; BMI, Body mass index.

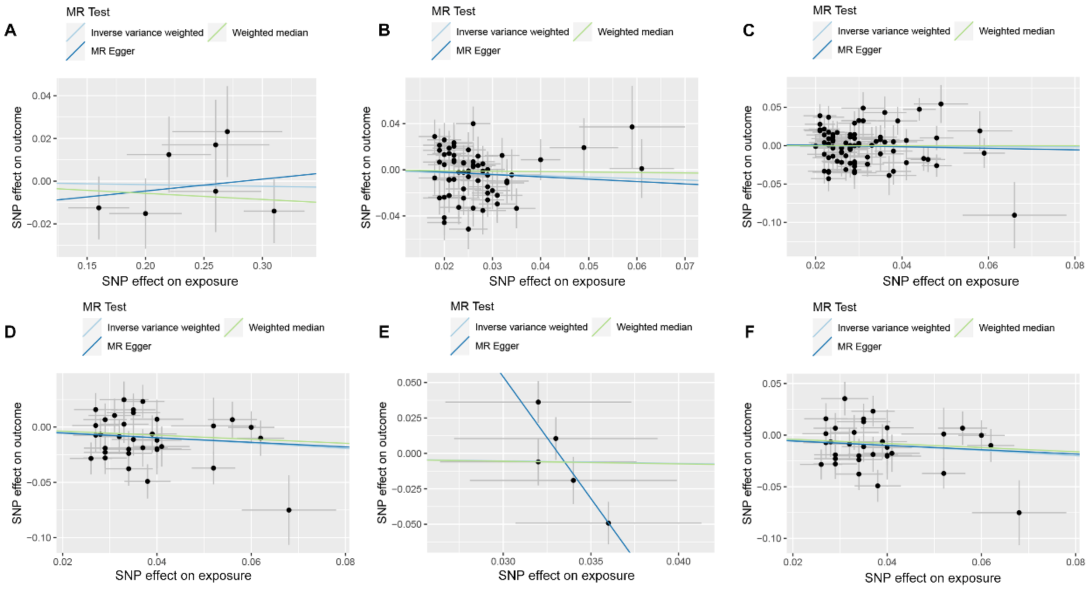

Figure S6. Scatterplots of the causal estimates of body fat distribution and Radial peak diastolic strain rate. Scatterplots of SNP effects on BMI (A), Waist circumference (B), Hip circumference (C), Waist-to-hip ratio (D), Waist-to-hip ratio for male (E), Waist-to-hip ratio for female (F) versus their effects on Radial peak diastolic strain rate, with the slope of each line corresponding to the estimated MR effect of inverse variance-weighted, Weighted median, and MR-Egger, respectively. Error bars indicate 95% CIs. SNPs, single nucleotide polymorphisms; LV, left ventricular; BMI, Body mass index.

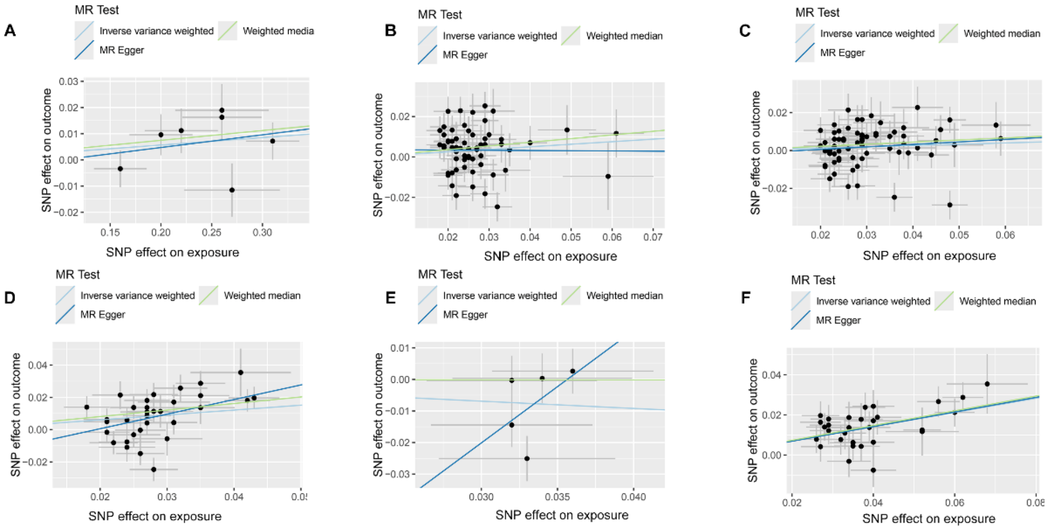

Figure S7. Scatterplots of the causal estimates of body fat distribution and left ventricular end diastole inferior wall thickness. Scatterplots of SNP effects on BMI (A), Waist circumference (B), Hip circumference (C), Waist-to-hip ratio (D), Waist-to-hip ratio for male (E), Waist-to-hip ratio for female (F) versus their effects on Radial peak diastolic strain rate, with the slope of each line corresponding to the estimated MR effect of inverse variance-weighted, Weighted median, and MR-Egger, respectively. Error bars indicate 95% CIs. SNPs, single nucleotide polymorphisms; LV, left ventricular; BMI, Body mass index.

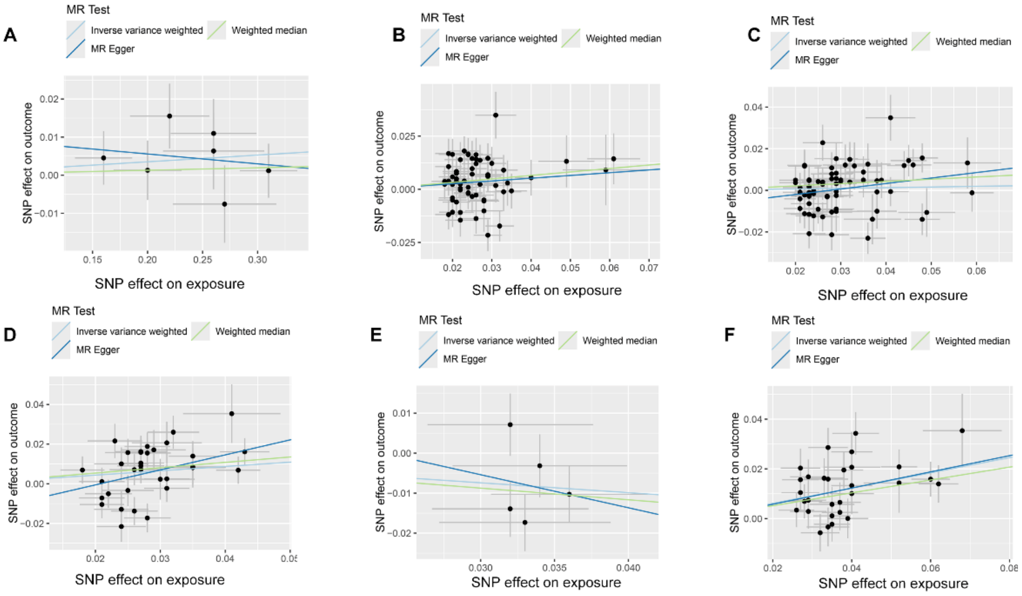

Figure S8. Scatterplots of the causal estimates of body fat distribution and left ventricular end diastole inferolateral wall thickness. Scatterplots of SNP effects on BMI (A), Waist circumference (B), Hip circumference (C), Waist-to-hip ratio (D), Waist-to-hip ratio for male (E), Waist-to-hip ratio for female (F) versus their effects on Radial peak diastolic strain rate, with the slope of each line corresponding to the estimated MR effect of inverse variance-weighted, Weighted median, and MR-Egger, respectively. Error bars indicate 95% CIs. SNPs, single nucleotide polymorphisms; LV, left ventricular; BMI, Body mass index.

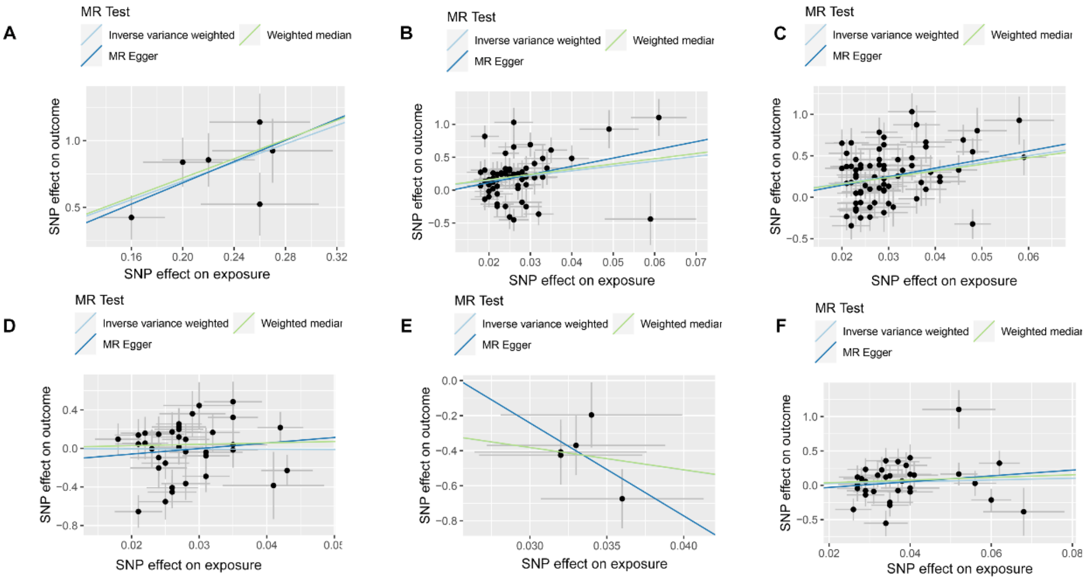

Figure S9. Scatterplots of the causal estimates of body fat distribution and left ventricular mass. Scatterplots of SNP effects on BMI (A), Waist circumference (B), Hip circumference (C), Waist-to-hip ratio (D), Waist-to-hip ratio for male (E), Waist-to-hip ratio for female (F) versus their effects on Radial peak diastolic strain rate, with the slope of each line corresponding to the estimated MR effect of inverse variance-weighted, Weighted median, and MR-Egger, respectively. Error bars indicate 95% CIs. SNPs, single nucleotide polymorphisms; LV, left ventricular; BMI, Body mass index.

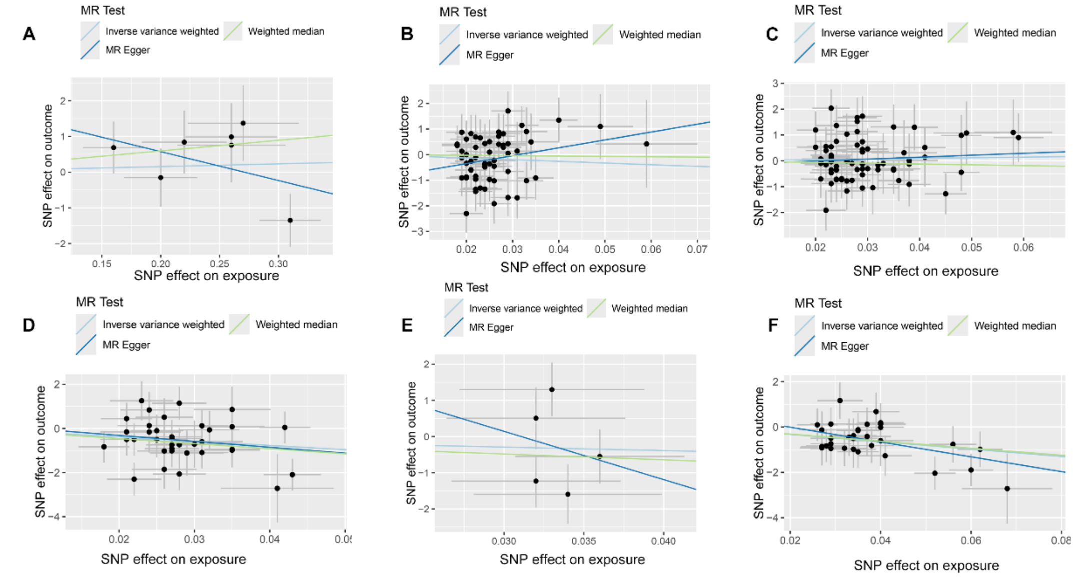

Figure S10. Scatterplots of the causal estimates of body fat distribution and left ventricular peak filling rate. Scatterplots of SNP effects on BMI (A), Waist circumference (B), Hip circumference (C), Waist-to-hip ratio (D), Waist-to-hip ratio for male (E), Waist-to-hip ratio for female (F) versus their effects on Radial peak diastolic strain rate, with the slope of each line corresponding to the estimated MR effect of inverse variance-weighted, Weighted median, and MR-Egger, respectively. Error bars indicate 95% CIs. SNPs, single nucleotide polymorphisms; LV, left ventricular; BMI, Body mass index.

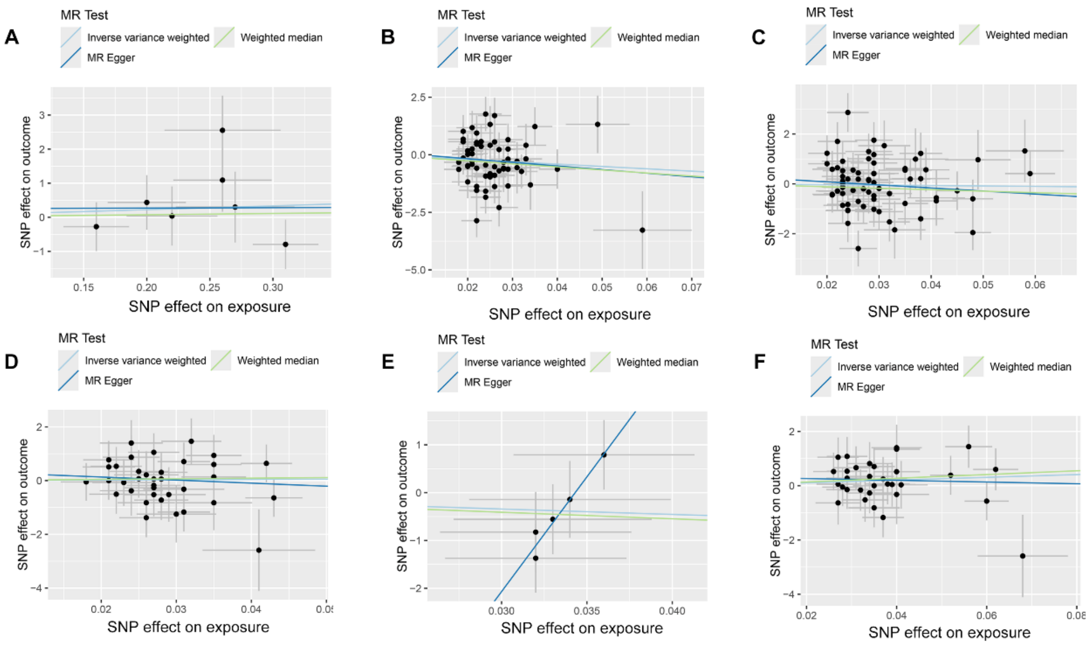

Figure S11. Scatterplots of the causal estimates of body fat distribution and left ventricular peak ejection rate. Scatterplots of SNP effects on BMI (A), Waist circumference (B), Hip circumference (C), Waist-to-hip ratio (D), Waist-to-hip ratio for male (E), Waist-to-hip ratio for female (F) versus their effects on Radial peak diastolic strain rate, with the slope of each line corresponding to the estimated MR effect of inverse variance-weighted, Weighted median, and MR-Egger, respectively. Error bars indicate 95% CIs. SNPs, single nucleotide polymorphisms; LV, left ventricular; BMI, Body mass index.

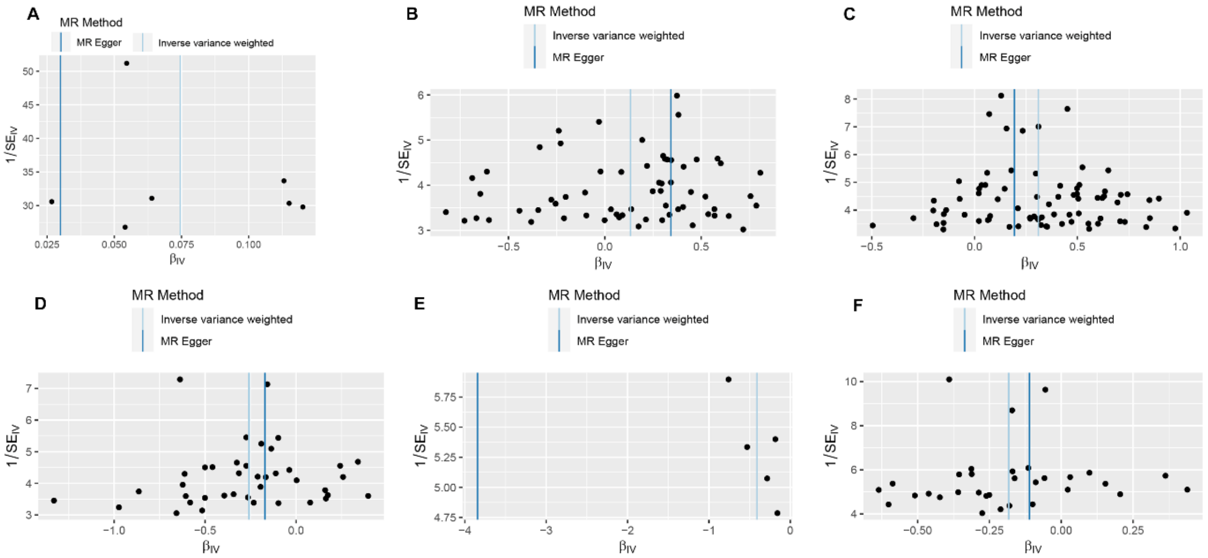

Figure S12. Funnel plots of the causal estimates of body fat distribution and left ventricular end-diastolic volume. Funnel plots of SNP effects on BMI (A), Waist circumference (B), Hip circumference (C), Waist-to-hip ratio (D), Waist-to-hip ratio for male (E), Waist-to-hip ratio for female (F) versus their effects on LV end-diastolic volume. SNPs, single nucleotide polymorphisms; LV, left ventricular; BMI, Body mass index.

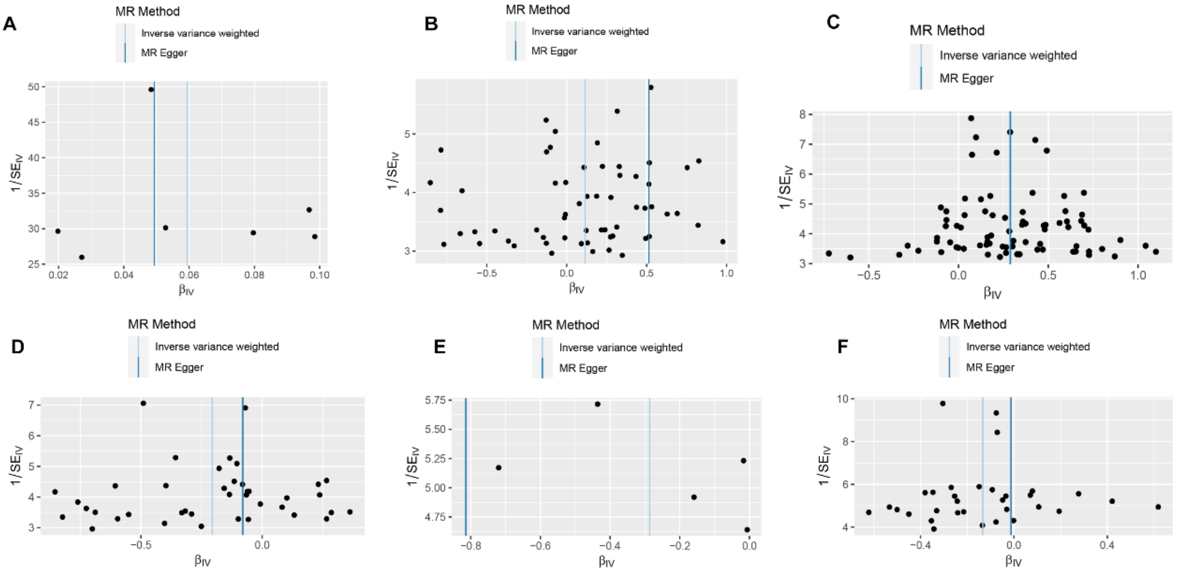

Figure S13. Funnel plots of the causal estimates of body fat distribution and left ventricular end-systolic volume. Funnel plots of SNP effects on BMI (A), Waist circumference (B), Hip circumference (C), Waist-to-hip ratio (D), Waist-to-hip ratio for male (E), Waist-to-hip ratio for female (F) versus their effects on LV end-systolic volume. SNPs, single nucleotide polymorphisms; LV, left ventricular; BMI, Body mass index.

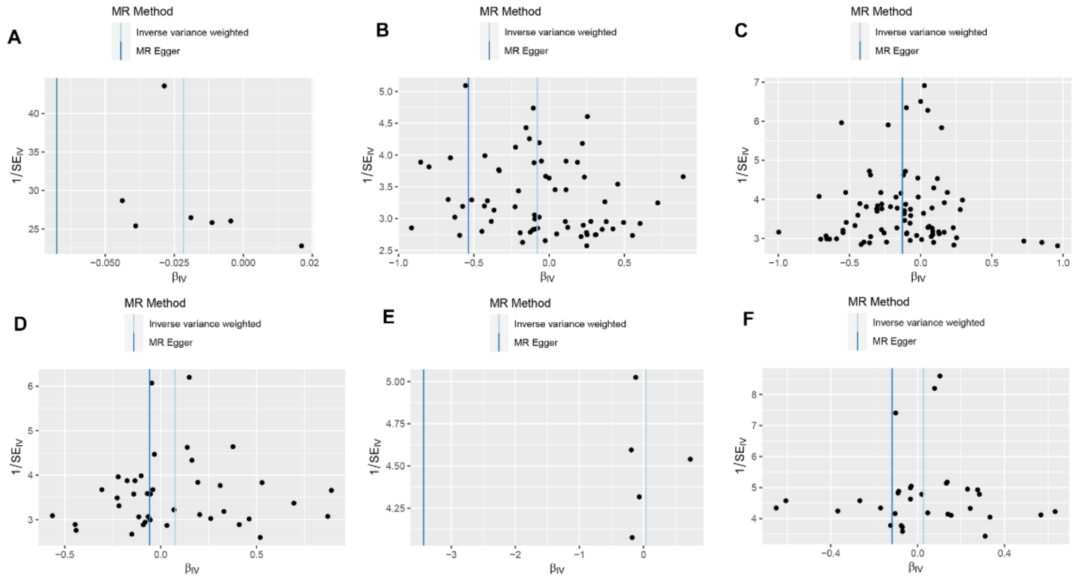

Figure S14. Funnel plots of the causal estimates of body fat distribution and left ventricular ejection fraction. Funnel plots of SNP effects on BMI (A), Waist circumference (B), Hip circumference (C), Waist-to-hip ratio (D), Waist-to-hip ratio for male (E), Waist-to-hip ratio for female (F) versus their effects on LV ejection fraction. SNPs, single nucleotide polymorphisms; LV, left ventricular; BMI, Body mass index.

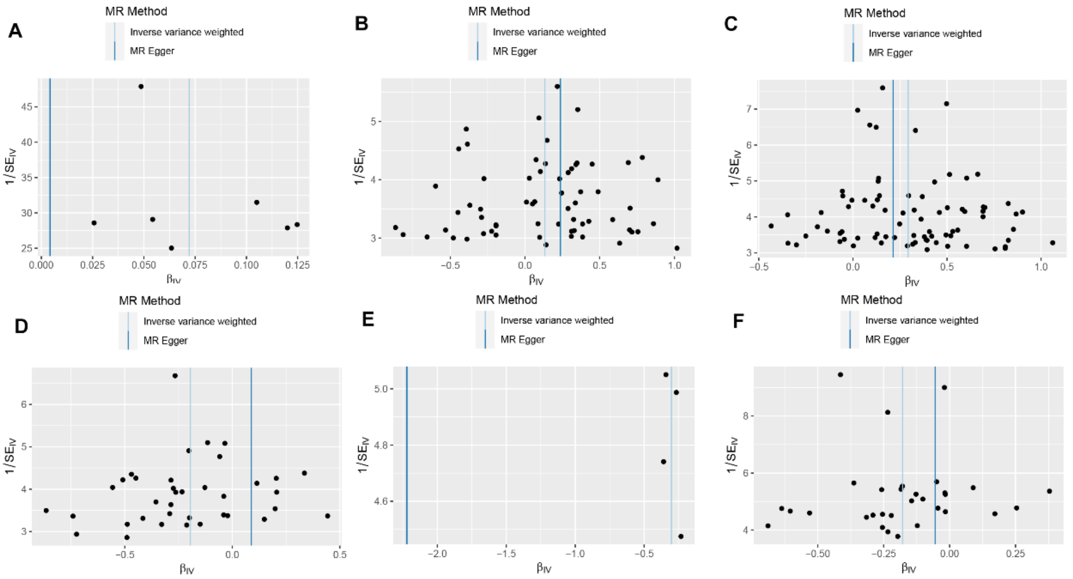

Figure S15. Funnel plots of the causal estimates of body fat distribution and left ventricular stroke volume. Funnel plots of SNP effects on BMI (A), Waist circumference (B), Hip circumference (C), Waist-to-hip ratio (D), Waist-to-hip ratio for male (E), Waist-to-hip ratio for female (F) versus their effects on LV stroke volume. SNPs, single nucleotide polymorphisms; LV, left ventricular; BMI, Body mass index.

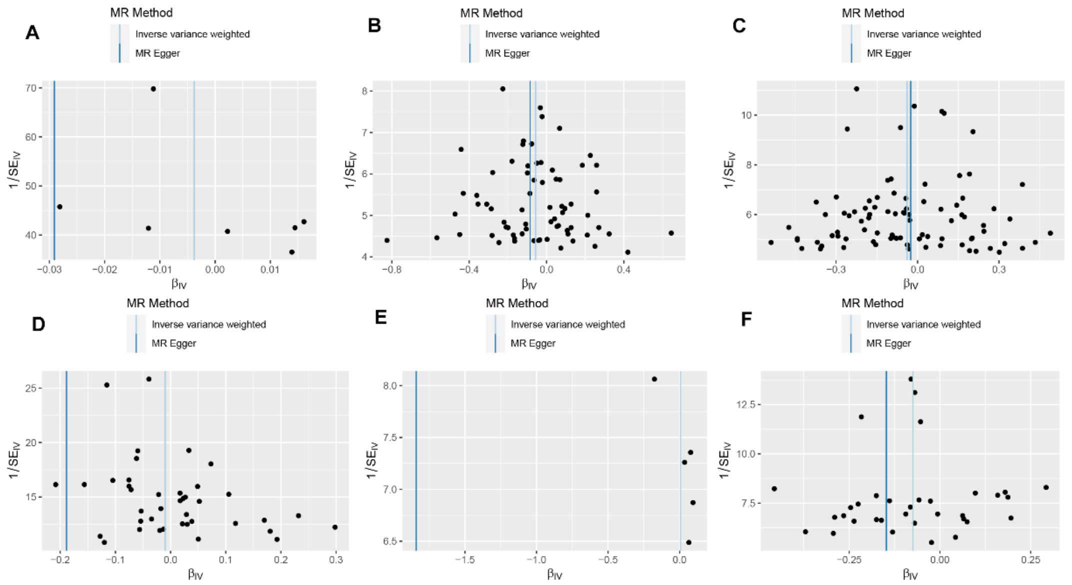

Figure S16. Funnel plots of the causal estimates of body fat distribution and Longitudinal peak diastolic strain rate. Funnel plots of SNP effects on BMI (A), Waist circumference (B), Hip circumference (C), Waist-to-hip ratio (D), Waist-to-hip ratio for male (E), Waist-to-hip ratio for female (F) versus their effects on Longitudinal peak diastolic strain rate. SNPs, single nucleotide polymorphisms; LV, left ventricular; BMI, Body mass index.

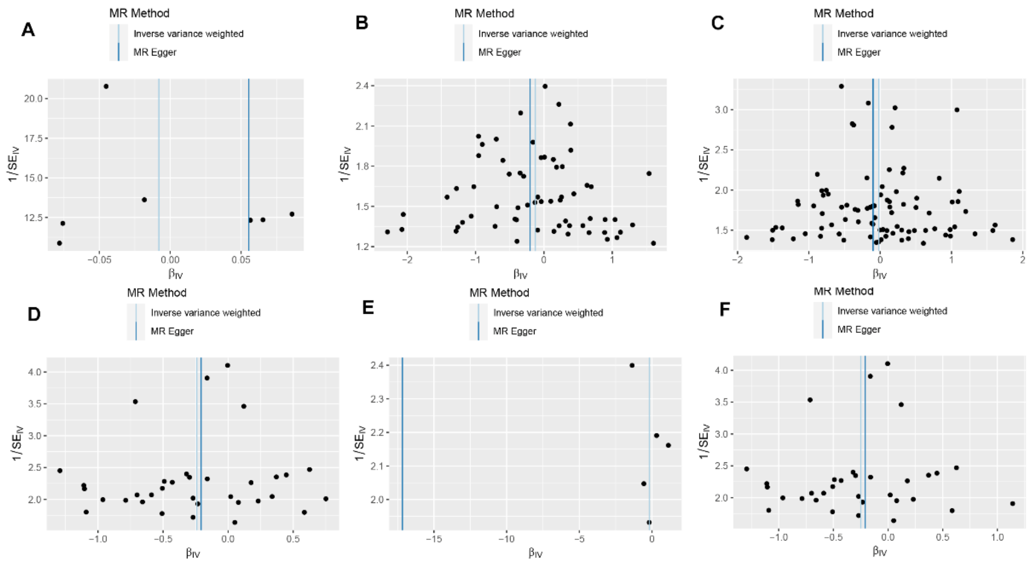

Figure S17. Funnel plots of the causal estimates of body fat distribution and Radial peak diastolic strain rate. Funnel plots of SNP effects on BMI (A), Waist circumference (B), Hip circumference (C), Waist-to-hip ratio (D), Waist-to-hip ratio for male (E), Waist-to-hip ratio for female (F) versus their effects on Radial peak diastolic strain rate. SNPs, single nucleotide polymorphisms; LV, left ventricular; BMI, Body mass index.

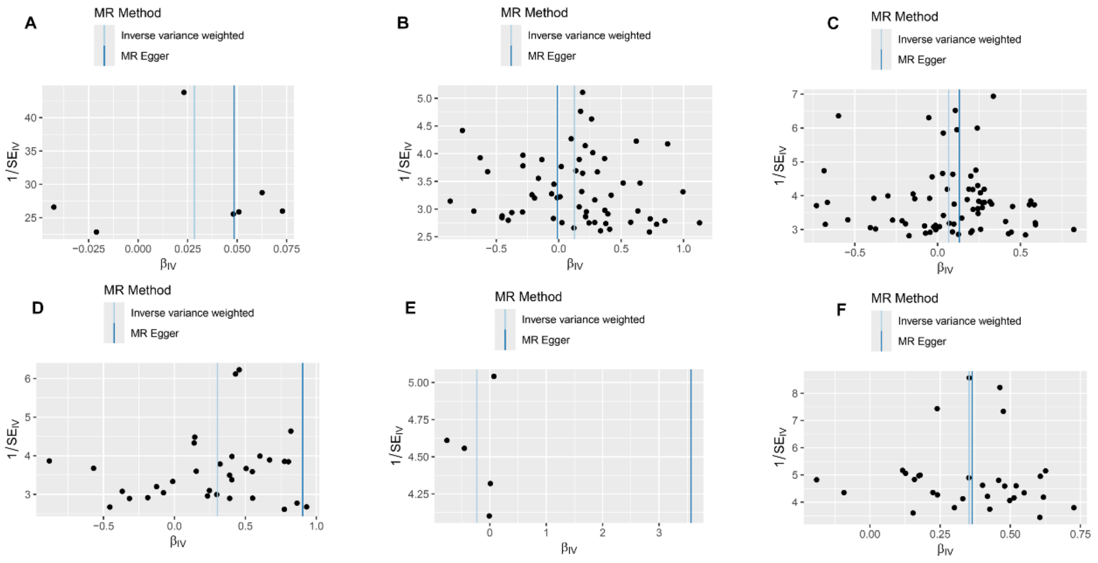

Figure S18. Funnel plots of the causal estimates of body fat distribution and left ventricular end diastole inferior wall thickness. Funnel plots of SNP effects on BMI (A), Waist circumference (B), Hip circumference (C), Waist-to-hip ratio (D), Waist-to-hip ratio for male (E), Waist-to-hip ratio for female (F) versus their effects on Radial peak diastolic strain rate. SNPs, single nucleotide polymorphisms; LV, left ventricular; BMI, Body mass index.

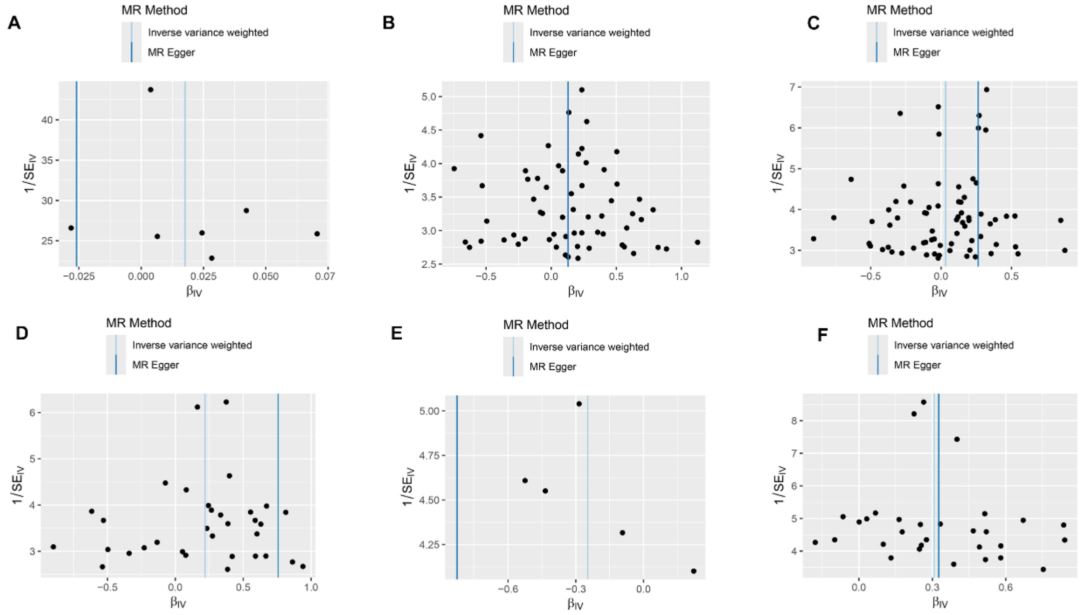

Figure S19. Funnel plots of the causal estimates of body fat distribution and left ventricular end diastole inferolateral wall thickness. Funnel plots of SNP effects on BMI (A), Waist circumference (B), Hip circumference (C), Waist-to-hip ratio (D), Waist-to-hip ratio for male (E), Waist-to-hip ratio for female (F) versus their effects on Radial peak diastolic strain rate. SNPs, single nucleotide polymorphisms; LV, left ventricular; BMI, Body mass index.

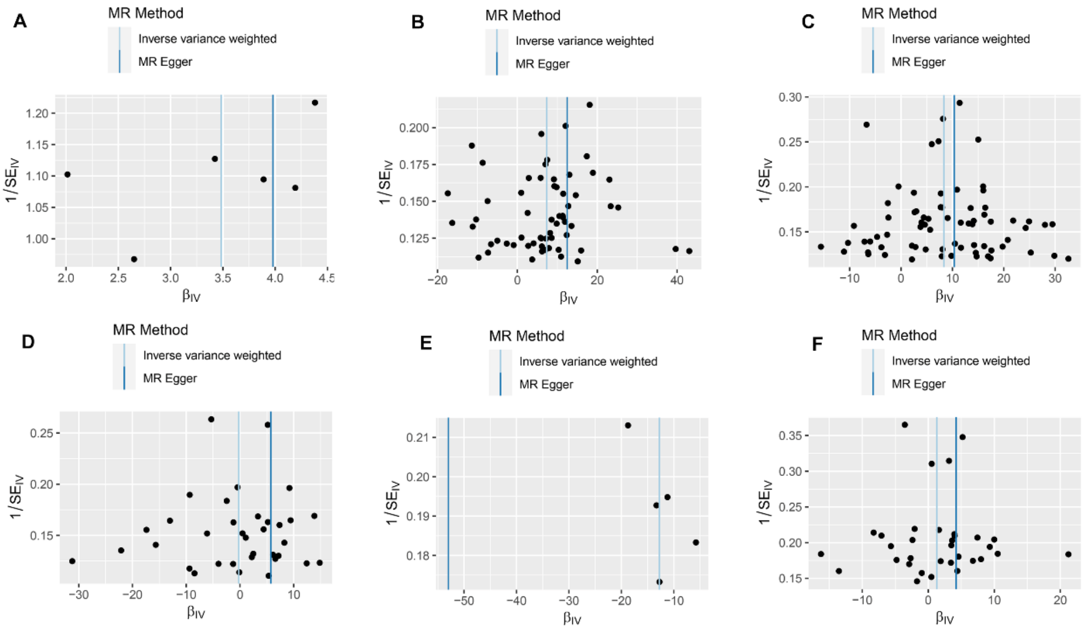

Figure S20. Funnel plots of the causal estimates of body fat distribution and left ventricular mass. Funnel plots of SNP effects on BMI (A), Waist circumference (B), Hip circumference (C), Waist-to-hip ratio (D), Waist-to-hip ratio for male (E), Waist-to-hip ratio for female (F) versus their effects on Radial peak diastolic strain rate. SNPs, single nucleotide polymorphisms; LV, left ventricular; BMI, Body mass index.

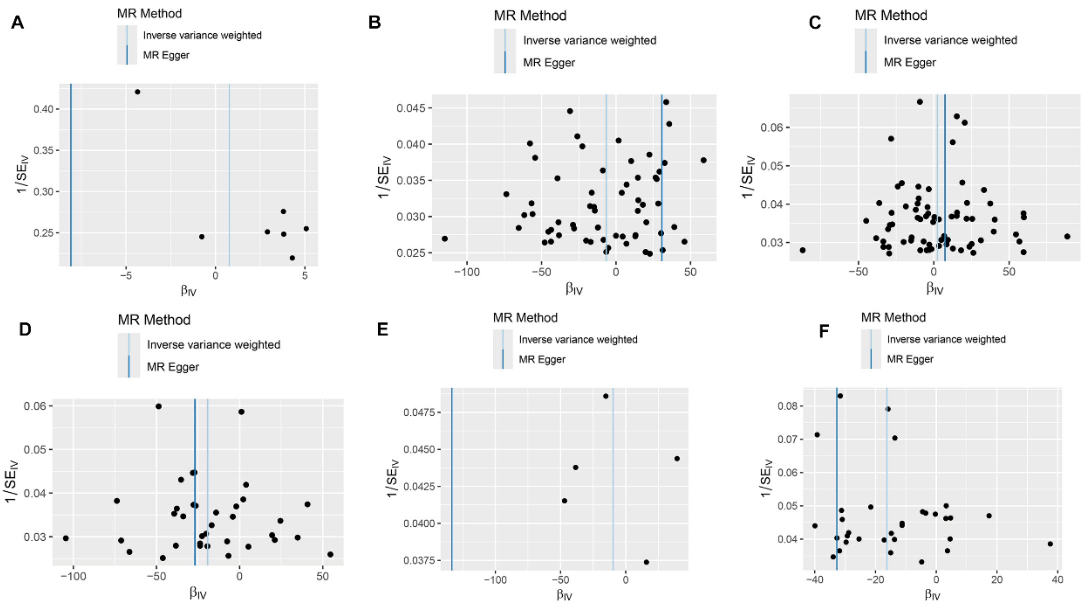

Figure S21. Funnel plots of the causal estimates of body fat distribution and left ventricular peak filling rate. Funnel plots of SNP effects on BMI (A), Waist circumference (B), Hip circumference (C), Waist-to-hip ratio (D), Waist-to-hip ratio for male (E), Waist-to-hip ratio for female (F) versus their effects on Radial peak diastolic strain rate. SNPs, single nucleotide polymorphisms; LV, left ventricular; BMI, Body mass index.

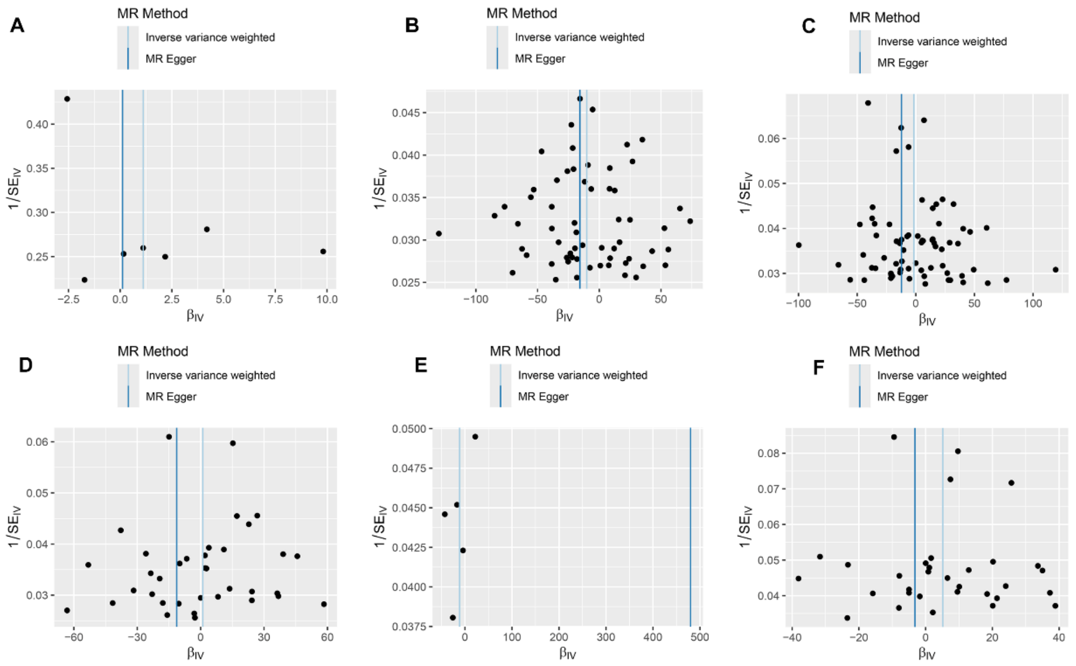

Figure S22. Funnel plots of the causal estimates of body fat distribution and left ventricular peak ejection rate. Funnel plots of SNP effects on BMI (A), Waist circumference (B), Hip circumference (C), Waist-to-hip ratio (D), Waist-to-hip ratio for male (E), Waist-to-hip ratio for female (F) versus their effects on Radial peak diastolic strain rate. SNPs, single nucleotide polymorphisms; LV, left ventricular; BMI, Body mass index.

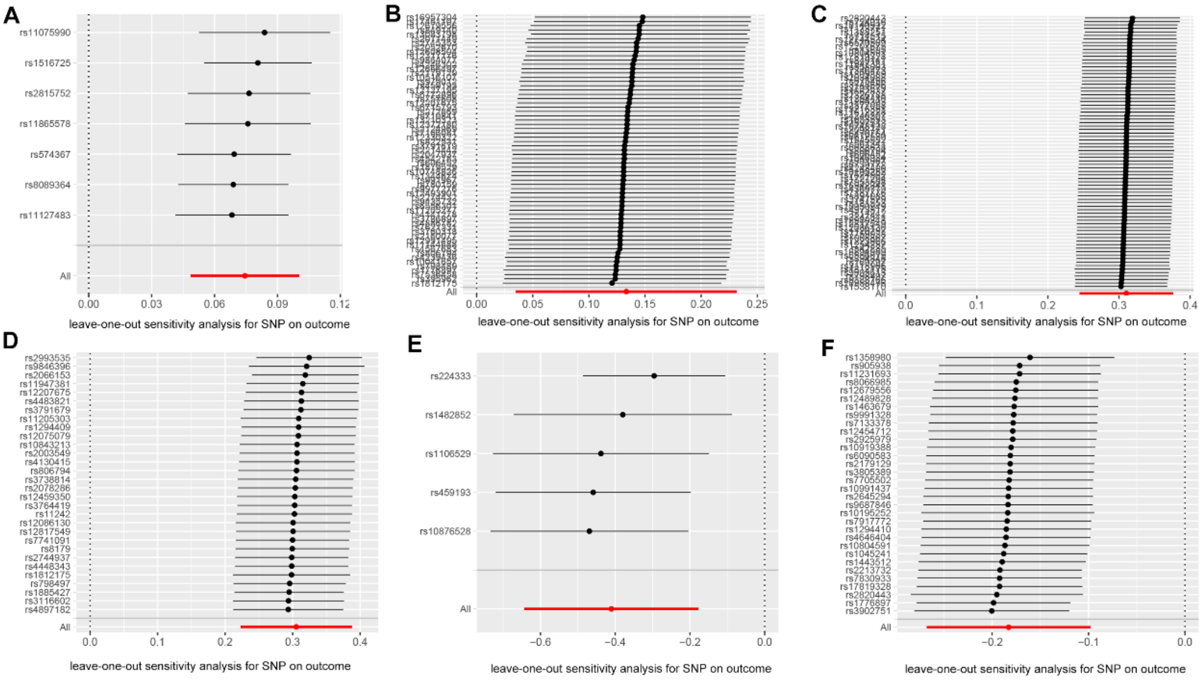

Figure S23. Leave-one-out analyses of the association between body fat distribution and left ventricular end-diastolic volume. Leave-one-out analyses of SNP effects on BMI (A), Waist circumference (B), Hip circumference (C), Waist-to-hip ratio (D), Waist-to-hip ratio for male (E), Waist-to-hip ratio for female (F) versus their effects on LV end-diastolic volume. SNPs, single nucleotide polymorphisms; LV, left ventricular; BMI, Body mass index.

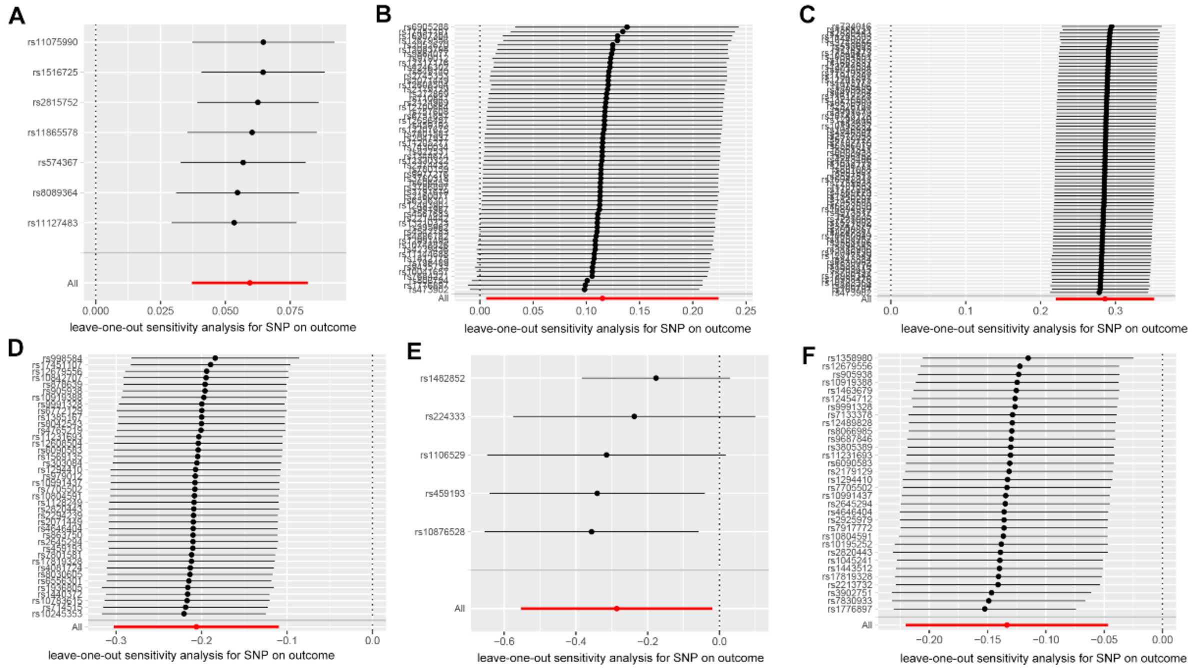

Figure S24. Leave-one-out analyses of the association between body fat distribution and left ventricular end- systolic volume. Leave-one-out analyses of SNP effects on BMI (A), Waist circumference (B), Hip circumference (C), Waist-to-hip ratio (D), Waist-to-hip ratio for male (E), Waist-to-hip ratio for female (F) versus their effects on LV end-systolic volume. SNPs, single nucleotide polymorphisms; LV, left ventricular; BMI, Body mass index.

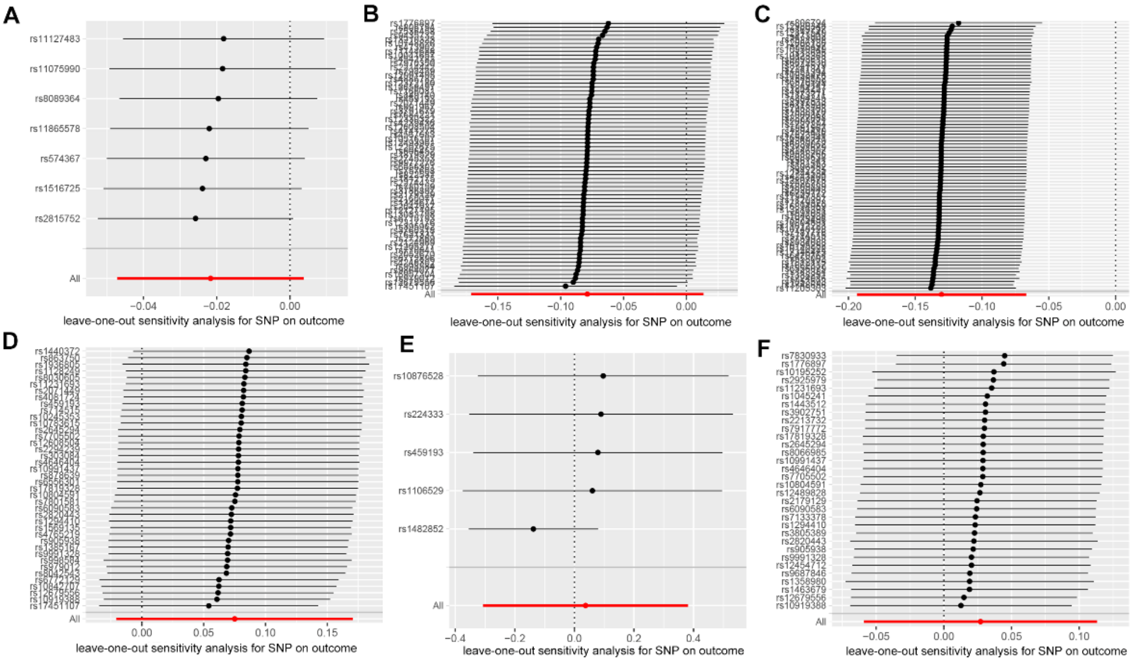

Figure S25. Leave-one-out analyses of the association between body fat distribution and left ventricular ejection fraction. Leave-one-out analyses of SNP effects on BMI (A), Waist circumference (B), Hip circumference (C), Waist-to-hip ratio (D), Waist-to-hip ratio for male (E), Waist-to-hip ratio for female (F) versus their effects on LV ejection fraction. SNPs, single nucleotide polymorphisms; LV, left ventricular; BMI, Body mass index.

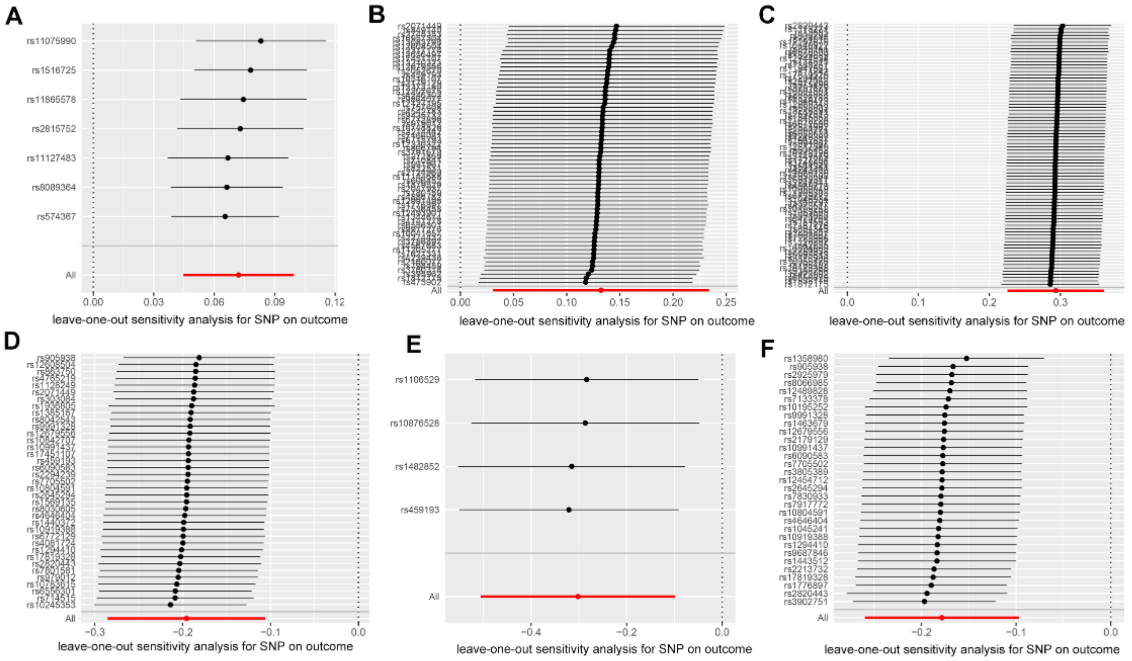

Figure S26. Leave-one-out analyses of the association between body fat distribution and left ventricular stroke volume. Leave-one-out analyses of SNP effects on BMI (A), Waist circumference (B), Hip circumference (C), Waist-to-hip ratio (D), Waist-to-hip ratio for male (E), Waist-to-hip ratio for female (F) versus their effects on LV stroke volume. SNPs, single nucleotide polymorphisms; LV, left ventricular; BMI, Body mass index.

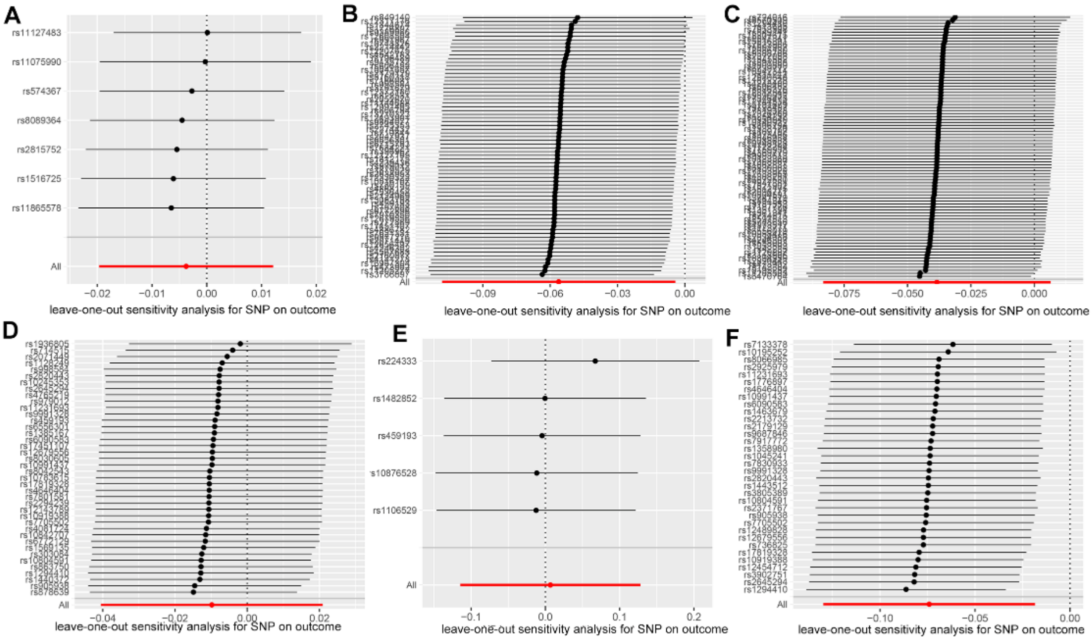

Figure S27. Leave-one-out analyses of the association between body fat distribution and Longitudinal peak diastolic strain rate. Leave-one-out analyses of SNP effects on BMI (A), Waist circumference (B), Hip circumference (C), Waist-to-hip ratio (D), Waist-to-hip ratio for male (E), Waist-to-hip ratio for female (F) versus their effects on Longitudinal peak diastolic strain rate. SNPs, single nucleotide polymorphisms; LV, left ventricular; BMI, Body mass index.

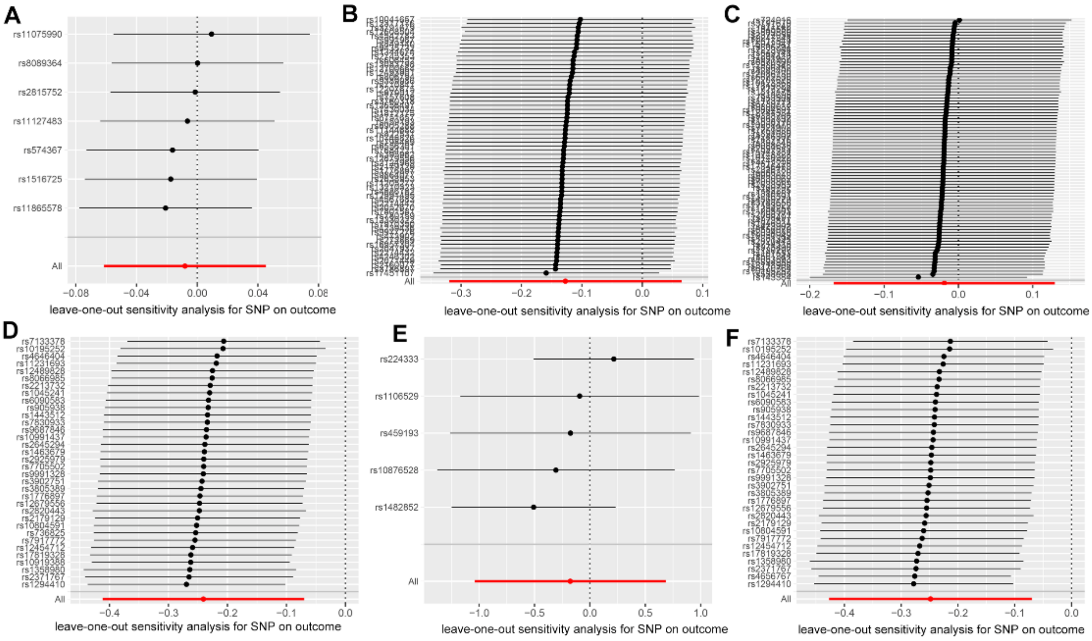

Figure S28. Leave-one-out analyses of the association between body fat distribution and Radial peak diastolic strain rate. Leave-one-out analyses of SNP effects on BMI (A), Waist circumference (B), Hip circumference (C), Waist-to-hip ratio (D), Waist-to-hip ratio for male (E), Waist-to-hip ratio for female (F) versus their effects on Radial peak diastolic strain rate. SNPs, single nucleotide polymorphisms; LV, left ventricular; BMI, Body mass index.

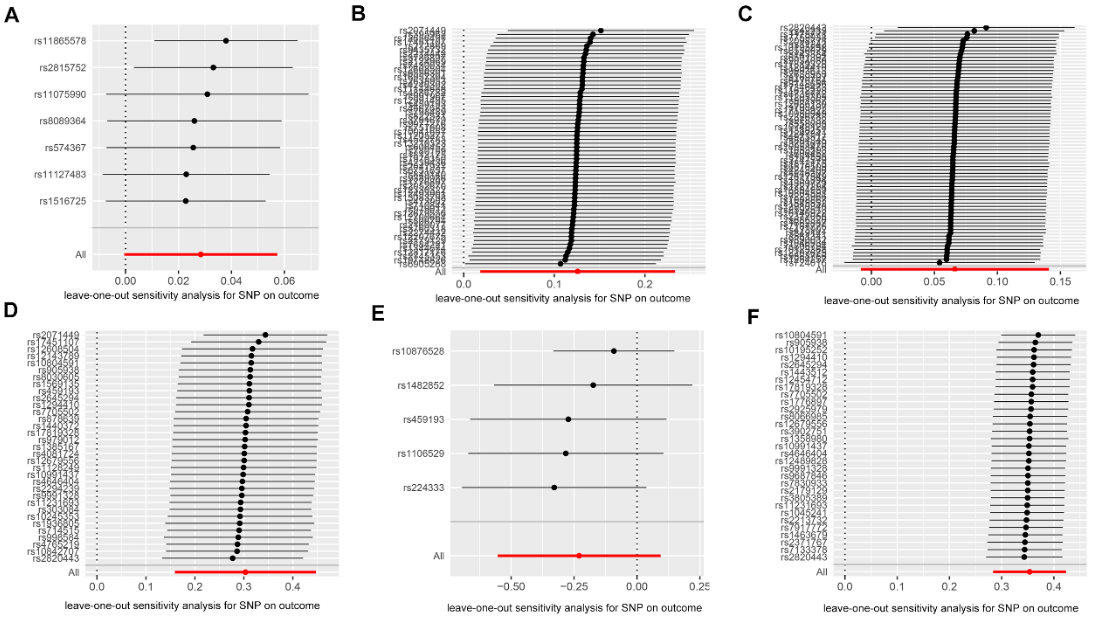

Figure S29. Leave-one-out analyses of the association between body fat distribution and left ventricular end diastole inferior wall thickness. Leave-one-out analyses of SNP effects on BMI (A), Waist circumference (B), Hip circumference (C), Waist-to-hip ratio (D), Waist-to-hip ratio for male (E), Waist-to-hip ratio for female (F) versus their effects on Radial peak diastolic strain rate. SNPs, single nucleotide polymorphisms; LV, left ventricular; BMI, Body mass index.

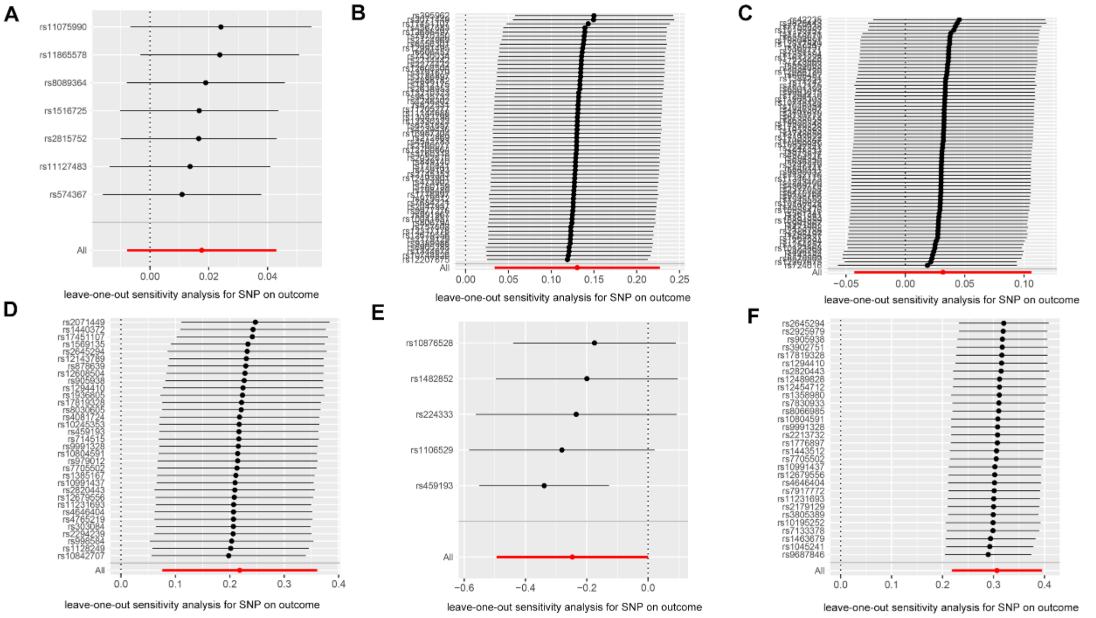

Figure S30. Leave-one-out analyses of the association between body fat distribution and left ventricular end diastole inferolateral wall thickness. Leave-one-out analyses of SNP effects on BMI (A), Waist circumference (B), Hip circumference (C), Waist-to-hip ratio (D), Waist-to-hip ratio for male (E), Waist-to-hip ratio for female (F) versus their effects on Radial peak diastolic strain rate. SNPs, single nucleotide polymorphisms; LV, left ventricular; BMI, Body mass index.

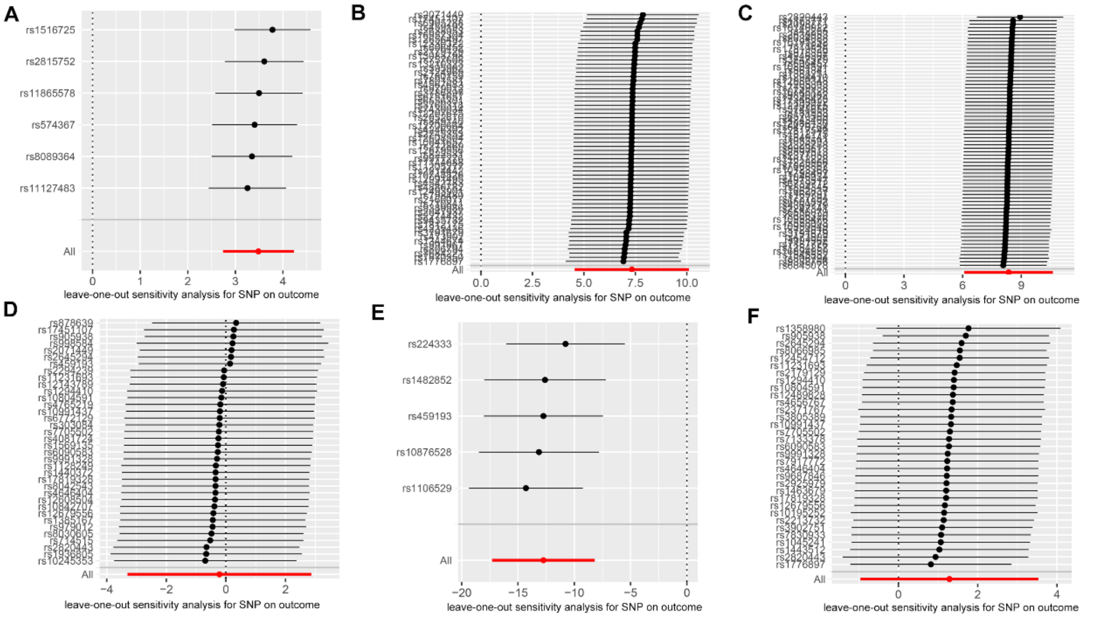

Figure S31. Leave-one-out analyses of the association between body fat distribution and left ventricular mass. Leave-one-out analyses of SNP effects on BMI (A), Waist circumference (B), Hip circumference (C), Waist-to-hip ratio (D), Waist-to-hip ratio for male (E), Waist-to-hip ratio for female (F) versus their effects on Radial peak diastolic strain rate. SNPs, single nucleotide polymorphisms; LV, left ventricular; BMI, Body mass index.

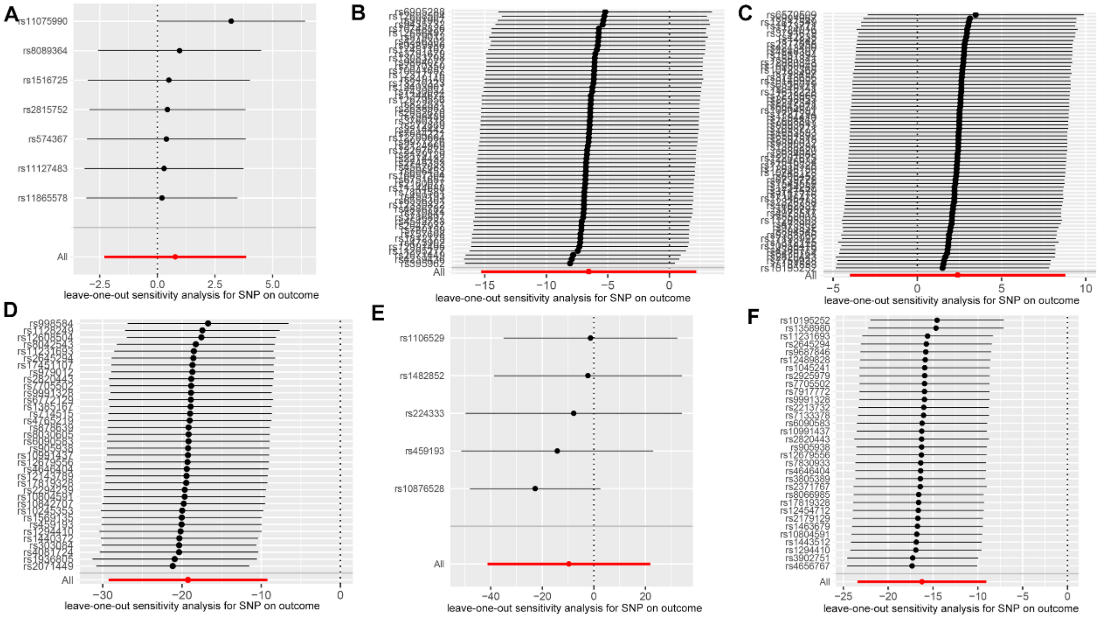

Figure S32. Leave-one-out analyses of the association between body fat distribution and left ventricular peak filling rate. Leave-one-out analyses of SNP effects on BMI (A), Waist circumference (B), Hip circumference (C), Waist-to-hip ratio (D), Waist-to-hip ratio for male (E), Waist-to-hip ratio for female (F) versus their effects on Radial peak diastolic strain rate. SNPs, single nucleotide polymorphisms; LV, left ventricular; BMI, Body mass index.

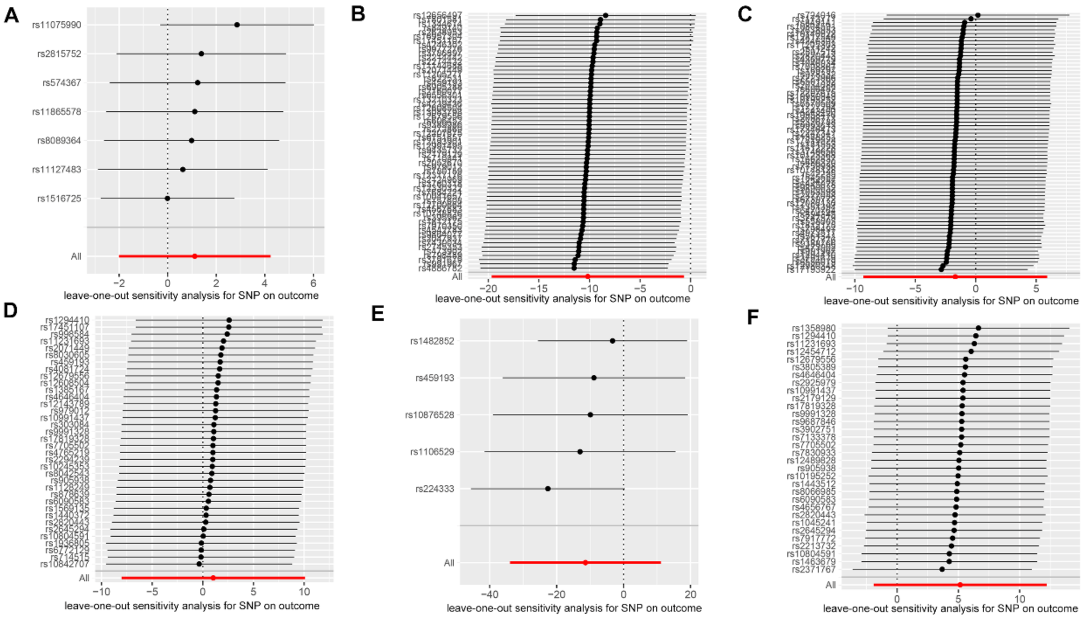

Figure S33. Leave-one-out analyses of the association between body fat distribution and left ventricular peak ejection rate. Leave-one-out analyses of SNP effects on BMI (A), Waist circumference (B), Hip circumference (C), Waist-to-hip ratio (D), Waist-to-hip ratio for male (E), Waist-to-hip ratio for female (F) versus their effects on Radial peak diastolic strain rate. SNPs, single nucleotide polymorphisms; LV, left ventricular; BMI, Body mass index.
